# RUNX1-ETO Depletion in t(8;21) AML Leads to C/EBPα- and AP-1-Mediated Alterations in Enhancer-Promoter Interaction

**DOI:** 10.1016/j.celrep.2019.08.040

**Published:** 2019-09-17

**Authors:** Anetta Ptasinska, Anna Pickin, Salam A. Assi, Paulynn Suyin Chin, Luke Ames, Roberto Avellino, Stefan Gröschel, Ruud Delwel, Peter N. Cockerill, Cameron S. Osborne, Constanze Bonifer

**Affiliations:** 1 Institute of Cancer and Genomic Sciences, University of Birmingham, Birmingham B152TT, UK; 2 Department of Medical & Molecular Genetics, King’s College London, London SE1 9RT, UK; 3 Department of Hematology, Erasmus University Medical Center, Rotterdam, the Netherlands; 4 Oncode Institute, Erasmus University Medical Center, Rotterdam, the Netherlands

**Keywords:** acute myeloid leukemia, RUNX1-ETO, promoter-enhancer interactions, Promoter-Capture Hi-C, transcriptional networks, chromatin programming, transcription factors, epigenetic regulation, integrated analysis of high-throughput data, AP-1 signaling in acute myeloid leukemia

## Abstract

Acute myeloid leukemia (AML) is associated with mutations in transcriptional and epigenetic regulator genes impairing myeloid differentiation. The t(8;21)(q22;q22) translocation generates the RUNX1-ETO fusion protein, which interferes with the hematopoietic master regulator RUNX1. We previously showed that the maintenance of t(8;21) AML is dependent on RUNX1-ETO expression. Its depletion causes extensive changes in transcription factor binding, as well as gene expression, and initiates myeloid differentiation. However, how these processes are connected within a gene regulatory network is unclear. To address this question, we performed Promoter-Capture Hi-C assays, with or without RUNX1-ETO depletion and assigned interacting cis-regulatory elements to their respective genes. To construct a RUNX1-ETO-dependent gene regulatory network maintaining AML, we integrated cis-regulatory element interactions with gene expression and transcription factor binding data. This analysis shows that RUNX1-ETO participates in cis-regulatory element interactions. However, differential interactions following RUNX1-ETO depletion are driven by alterations in the binding of RUNX1-ETO-regulated transcription factors.

## Highlights

• Promoter Capture Hi-C links t(8;21) AML-specific cis-elements to correct promoters
• Interacting cis-elements bound by transcription factors form a gene regulatory network
• The product of the t(8;21) translocation, RUNX1-ETO, participates in interactions
• Differential interactions after RUNX1-ETO depletion are driven by C/EBPα and AP-1

## Graphical Abstract

**Figure:**
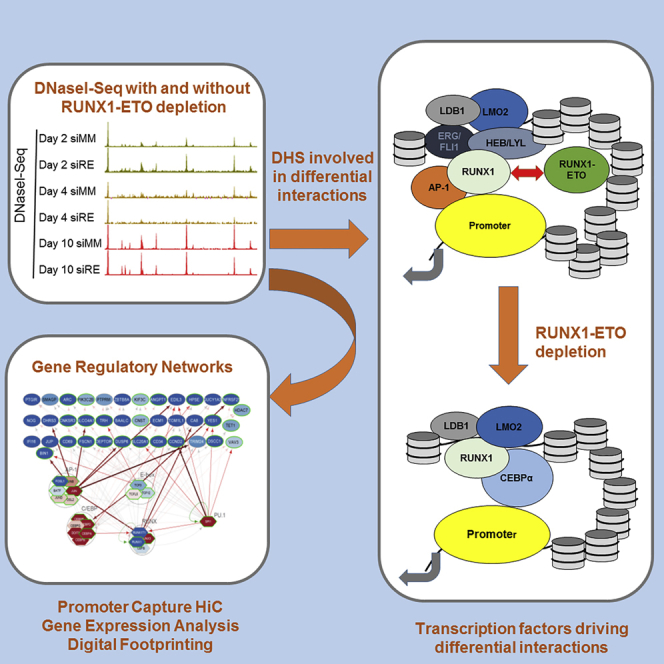

## Teaser

Promoter-Capture Hi-C assays, gene expression, and transcription-factor binding data are used to construct a RUNX1-ETO-dependent dynamic gene regulatory network that maintains acute myeloid leukemia (AML). Ptasinska et al. show that RUNX1-ETO participates in cis-regulatory element interactions and that differential interactions after RUNX1-ETO depletion are driven by C/EBPα and AP-1.

## Introduction

Acute myeloid leukemia (AML) is a hematopoietic malignancy caused by genetic abnormalities in hematopoietic stem cells (HSCs), which restrict their ability to undergo the normal differentiation process (Bonifer and Cockerill, 2015, Kumar, 2011). The transcription factors (TFs) regulating hematopoiesis have to be expressed in a stage- and lineage-restricted fashion since their mutation or de-regulation impairs differentiation and prolongs the proliferative stage, thus increasing the opportunities for cells to further mutate and progress to AML (Bonifer and Cockerill, 2011, Rosenbauer and Tenen, 2007). One of the best-studied subtypes of AML is the t(8;21)(q22;q22) translocation generating the RUNX1-ETO fusion protein (Erickson et al., 1992, Miyoshi et al., 1991). RUNX1-ETO has a modular structure comprising the RUNX1 DNA-binding domain plus four evolutionary conserved functional domains named nervy homology regions 1 to 4 (NHR1 to NHR4) (Kitabayashi et al., 1998), which recruit transcriptional repressors such as the N-CoR/mSin3/HDAC1 complex (Lutterbach et al., 1998). The expression of this abnormal protein results in a block in differentiation and increased cell survival (Dunne et al., 2006, Heidenreich et al., 2003, Martinez et al., 2004, Ptasinska et al., 2012).

The RUNX1-ETO fusion protein is part of a larger TF complex consisting of RUNX1-ETO; CBFβ; the erythroblast transformation-specific (ETS) family of transcription factors (ERG and FLI1); E proteins such as HEB, E2A, and LYL1; and the non-DNA binding factors LDB1 and LMO2 (Martens et al., 2012, Ptasinska et al., 2014, Sun et al., 2013). Each part of the complex is thought to be essential for AML maintenance (Sun et al., 2013). RUNX1-ETO depletion in t(8;21) cells is sufficient to trigger extensive global changes in the transcriptomic and epigenetic profile across hundreds of genes (Ben-Ami et al., 2013, Dunne et al., 2006, Ptasinska et al., 2012, Sun et al., 2013). Depletion upregulates a specific set of RUNX1-regulated genes, such as *CEBPA*, leading to increased recruitment of RUNX1 and C/EBPα to gene regulatory elements throughout the genome, thereby releasing the block on myeloid differentiation and suppressing self-renewal (Loke et al., 2017, Ptasinska et al., 2014, Sun et al., 2013). We have previously used global TF binding and gene expression information to construct a dynamic gene regulatory network linking genes bound by the RUNX1-ETO complex to dynamic changes of gene expression (Ptasinska et al., 2014). We used such system-wide information to devise a RNAi dropout screen that identified a number of genes associated with AML maintenance (Martinez-Soria et al., 2019). However, to fully explore the power of genome-wide studies, we need to construct gene regulatory networks that enable us to predict the results of perturbation experiments from such data using modeling approaches. Therefore, a number of issues still need to be resolved. RUNX1-ETO mostly binds to distal cis-regulatory elements, and although we can define genes responding to RUNX1-ETO knockdown, we do not know whether this response is direct or indirect, as we do not know which promoter is linked to the sites of fusion protein binding. In addition, we do not know which other TFs participate in the maintenance of the leukemic state and drive the response to RUNX1-ETO knockdown.

To answer these questions, we identified direct cis-element interactions using the Promoter Capture Hi-C (CHi-C) method (Mifsud et al., 2015) in Kasumi-1 cells, a well-known model of t(8;21) AML, with and without small interfering RNA (siRNA)-mediated RUNX1-ETO depletion. RUNX1-ETO knockdown leads to a rewiring of promoter-enhancer interactions, which is driven by increased C/EBPα and loss of AP-1 binding after knockdown. We integrated these results with chromatin immunoprecipitation (ChIP) and digital footprinting data from cell lines and patients to identify regulatory relationships between binding events and gene expression, which will aid further studies aimed at identifying pathways required for t(8;21) AML leukemic maintenance.

## Results and Discussion

### RUNX1-ETO Depletion Does Not Lead to a Global Reorganization of Chromosome Structure but Changes Promoter-Enhancer Interactions within TADs

**Figure 1:**
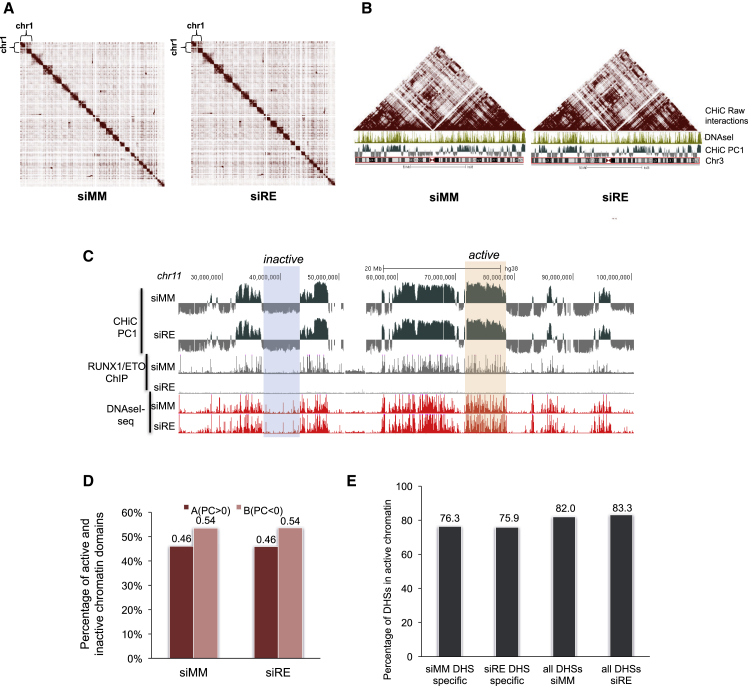
RUNX1-ETO and the Genome Organization in t(8;21) AML (A) Contact matrix across the whole genome. Each pixel represents a 10-Mb section of the genome. Color intensity represents interaction frequency. The left-hand plot shows a Capture HiC interaction matrix generated with data from Kasumi-1 cells transfected with mismatch control siRNA (siMM) for 4 days; the right-hand plots shows an interaction matrix from RUNX1-ETO-depleted Kasumi-1 cells transfected with the specific siRNA (siRE). (B) Contact matrix across chromosome 3 at 10-Mb resolution. The heatmap shows the raw interactions on chromosome 3 using Kasumi-1 cells transfected with siMM (left) and siRE (right); a UCSC track highlighting the DHS pattern is shown below each heatmap together with the CHi-C first principle component (PC1) plot (see below). (C) UCSC genome browser screenshot shows a first principle component plot for Capture HiC siMM and siRE samples plotted along with RUNX1-ETO ChIP data (Ptasinska et al., 2014) and DNaseI-seq control (siMM) and knockdown (siRE) data from Kasumi-1 cells for a 70-Mb regions on chromosome 11. (D) Percentage of DHSs in active and inactive chromatin compartments in Kasumi-1 cells transfected with siMM and siRE. (E) Percentage of DHSs found at day 10 of knockdown participating in promoter-enhancer interactions (determined at day 4 of knockdown) detected in all active chromatin regions of siMM cells or siRE cells (right two panels), and specific to siMM or siRE cells (left two panels), indicating that the majority of specific DHSs are already present at day 4.

The tissue specificity of gene expression is controlled by lineage-restricted TFs binding to distal cis-regulatory elements that need to physically interact with their target promoters in order to activate gene expression (de Laat and Duboule, 2013, Plank and Dean, 2014). To examine whether RUNX1-ETO influences genome-wide cis-element interactions, we generated duplicate CHi-C libraries (Mifsud et al., 2015) from Kasumi-1 cells that were either untreated (mismatch control siRNA [siMM]) or following a 4-day siRNA-mediated treatment to knockdown RUNX1-ETO (siRE) (Figure 1A). Data analysis of the sequenced libraries identified 57,775 significant interactions between promoters and distal elements before and 60,681 significant interactions after RUNX1-ETO depletion. CHi-C libraries were highly reproducible with an average of 70% overlap of significant interactions between replicates (Figures S1A–S1D). To align our CHi-C data with the coordinates of cis-regulatory elements, we mapped Deoxyribonuclease I (DNaseI) hypersensitive sites (DHSs) during a knockdown time course (Figure S1E). The presence (siMM) or absence (siRE) of RUNX1-ETO did not influence global chromosomal organization across all chromosomes (Figure 1A), including the organization of this region into topologically associated domains (TADs; large triangles, projected above the DHS pattern) (Gonzalez-Sandoval and Gasser, 2016; Figure 1B). Figure 1C shows a University of California, Santa Cruz (UCSC) genome browser screenshot highlighting active and inactive chromatin compartments plotted alongside RUNX1-ETO ChIP data (Ptasinska et al., 2012) and day-10 DNase I hypersensitive sites sequencing (DNaseI-seq) data (this manuscript). These analyses revealed clusters of interactions within active and inactive regions whose ratio was invariant even after an extended period of RUNX1-ETO depletion (Figure 1D).

To investigate whether specific DHS patterns seen following RUNX1-ETO depletion correlated with a specific stage of myeloid differentiation, we compared DNaseI data from control and RUNX1-ETO-depleted Kasumi-1 cells (days 2, 4, and 10) to published assay for transposase-accessible chromatin using sequencing (ATAC-seq) data defining the open chromatin regions of normal stem and progenitor cells representing different developmental stages (Corces et al., 2016; Figure S1F). DHSs specific for control cells (bottom) aligned more closely with HSCs and early progenitors and showed increased AP-1 motif enrichment, whereas DHSs specific for RUNX1-ETO-depleted cells (top) aligned with those of monocytic cells and were enriched for C/EBP motifs. RUNX1-ETO depletion had a profound effect on gene expression with a large number of genes changing expression by day 10 (Figure S1G). Flow cytometry and principal component analyses of DNaseI-seq data revealed that Kasumi-1 cells gradually lose their stem cell markers (CD34 and CD117) while principal component and correlation clustering analyses of the DNaseI-seq data indicated that at day 10, but not yet at day 4, they had differentiated toward monocytic cells (Figures S1J and S1K). Phenotypic changes were accompanied by changes in protein and mRNA expression for a number of TFs visible already at day 2, in particular C/EBPα (Figures S1L and S1M), which is rapidly upregulated after knockdown. *JUN* mRNA was strongly downregulated during the first days of knockdown but then was strongly upregulated in concordance with its important role in regulating monocyte and/or macrophage-specific gene expression (Heinz et al., 2010). The expression of JUND protein was upregulated as well, but note that the DNA-binding activity of the AP-1 family of TFs is regulated by signaling-mediated phosphorylation (Bejjani et al., 2019).

**Figure 2:**
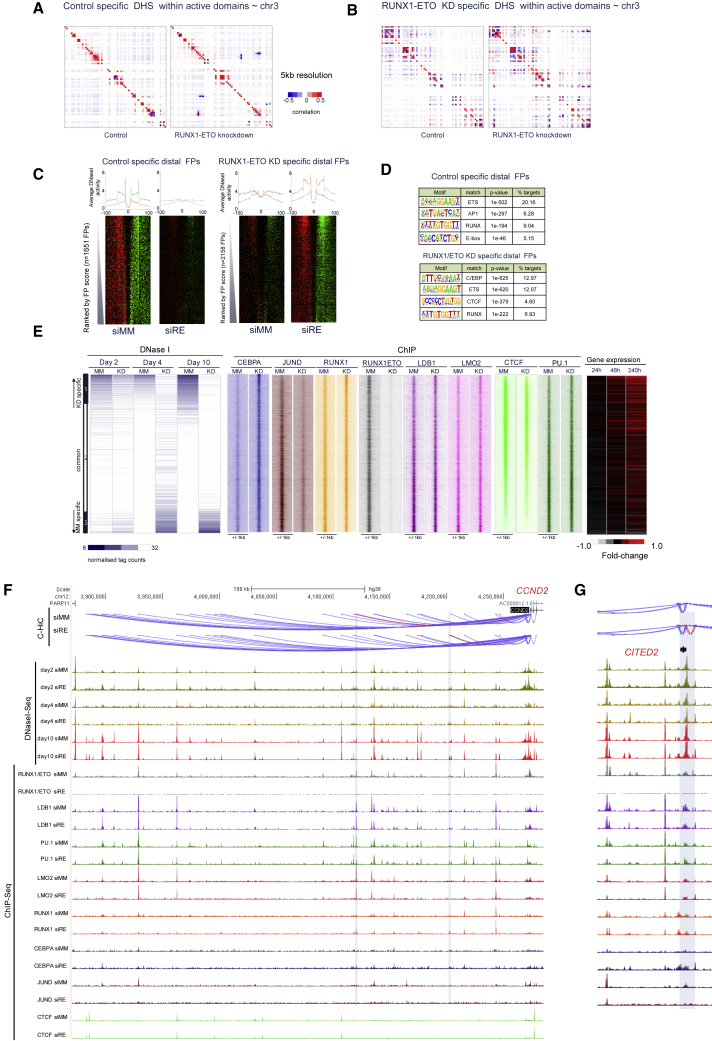
Differential Promoter-Enhancer Interactions after RUNX1-ETO Depletion Are Driven by Differential TF Binding (A) Heatmap representing the correlation of normalized interaction ratios across chr3 at 5-kb resolution, showing the correlation of CHiC peaks in regions specific to DHS peaks that are depleted after RUNX1-ETO knockdown. Each pixel represents a 5-kb section of the genome. The left panel shows the interaction heatmap for siMM and the right panel for siRE cells. Positive correlations are shown as red; negative correlation as blue squares. To determine statistically significant interactions, reads from replicates 1 and 2 were merged. (B) Heatmap representing the correlation of normalized interaction ratios across chr3 at 5-kb resolution and showing the correlation of CHi-C peaks in DHS peaks that are newly formed after RUNX1-ETO (R/E) gene knockdown. For all other features, see (A). (C) DNaseI cleavage patterns within specific distal footprints predicted by Wellington (Piper et al., 2013). Upper strand cut sites are shown in red and lower strand cut sites in green within a 200-bp window centered on each footprint (gap) for siMM- and siRE-specific footprints. (D) Analysis of overrepresented binding motifs within each footprint class as defined in (C). (E) Left panel: time course of DHS development after 2, 4, and 10 days of RUNX1-ETO depletion (see scheme in Figure S1E). Normalized tag counts are ranked alongside day-10 knockdown (KD) and control-specific (bottom) counts; common and siRE-specific DHS are indicated on the left. Alongside the same genomic coordinates, C/EBPα, JUND, LDB1, CTCF, RUNX1-ETO, LMO2, PU.1, and RUNX1 ChIP-seq reads from Kasumi-1 cells with or without RUNX1-ETO depletion are plotted as indicated (middle panel). The right panel shows the expression levels of the genes linked to the associated DNaseI-seq sites (right panel). (F) UCSC browser screenshot depicting interactions between the *CCND2* promoter and surrounding DHS (shown as arcs) together with the indicated ChIP-seq data before and after RUNX1-ETO knockdown. Changing interactions are shown in red, and their associated DHS/ChIP peaks are highlighted using a vertical shaded bar. (G) The same analysis as in (F) for the *CITED2* locus.

Around 80% of all DHSs detected in active regions of control cells or RUNX1-ETO-depleted cells participated in promoter-enhancer interactions (Figure 1E, right panels). To identify differential interactions, we used the CHi-C data to assign the respective DHSs to the promoter for RUNX1-ETO-depleted and control cells (Data S1). Figures 2A and 2B show statistically significant control-specific and RUNX1-ETO-specific interactions at 5-kb resolution involving specific DHSs on chromosome 3, which were not seen with shared DHSs (Figure S2A), indicating that it is the differential DHSs that drive these changes. A total of 1104 DHSs were significantly increased and 1209 were significantly decreased after 10 days of RUNX1-ETO knockdown (Figures 2E and S1F). The majority of these DHS (75% and 76%, respectively) show differential promoter-enhancer interactions already after day 4 of knockdown (Figure 1E), demonstrating that RUNX1-ETO depletion alters the epigenome prior to monocytic differentiation.

### RUNX1-ETO-Regulated TFs Drive Differential Cis-Regulatory Element Interactions

We next sought to identify the TFs driving the changes in interactions by performing digital footprinting analysis, using our Wellington algorithm (Piper et al., 2013). This approach reveals TF motifs protected from DNaseI digestion and evaluates genome-wide TF occupancy with high accuracy (Figure 2C). Examples of such footprints for day 10 of knockdown are depicted for ETS and C/EBP motifs at the *IL17RA* locus in Kasumi-1 cells (Figure S2B). Global binding motif analysis confirmed that AP-1 motifs were preferentially occupied in control (siMM) cells whereas C/EBP motifs were occupied in RUNX1-ETO-depleted (siRE) cells (Figure 2D). Motif occupancy was validated by comparing footprinting data with previously generated ChIP-seq data for C/EBPα, RUNX1-ETO, PU.1, JUND, and RUNX1 (Ptasinska et al., 2014, Martinez-Soria et al., 2019) (Figure S2C).

Two factors capable of mediating long-range interactions are CTCF and LDB1 (Deng et al., 2012, Splinter et al., 2006). To examine their role, we generated new CTCF and LDB1 ChIP data with and without RUNX1-ETO depletion. We correlated changes in gene expression and TF binding at specific DHSs with differential interactions between DHSs and promoters (Figure 2E). These analyses revealed a global increase in C/EBPα binding after knockdown and a decrease in JUND binding at siMM-specific DHSs (Figure 2E). All other factors showed no or little difference in binding between knockdown and control cells. Changing interactions correlated with differential gene expression (Figure 2E, outermost right panel). Figure 2F shows an example of interactions at the upregulated *CCND2* gene, which shows changes in interactions between its promoter and two upstream distal elements (depicted in red). Two observations are noteworthy. First, the *CCND2* promoter interacts with a large number of distal DHSs. Second, a large number of RUNX1-ETO binding sites are located within these sites, indicating that RUNX1-ETO is part of an extended and mostly invariant chromatin hub. To validate our Chi-C data, we conducted a circularized chromosome conformation capture (4C) experiment that investigated the *SPI1* (PU.1) locus at high resolution, using two different viewpoints (Figure S2D). We detected known interactions between the *SPI1* promoter and an upstream enhancer (URE) (Ebralidze et al., 2008), but also with two upstream promoters. These interactions did not change after RUNX1-ETO depletion. The same result was found using CHi-C (Figure S2E).

We next analyzed the behavior of LDB1 in more detail. LDB1 does not bind to DNA directly but via other TFs such as RUNX1 (Wadman et al., 1997). LDB1 binds to both promoter and distal regions (Figure S2E) and RUNX1-ETO depletion led to a loss of 1,506 LDB1 binding sites and the acquisition of 779 new sites (Figure S2F). De novo motif search of siMM- or siRE-specific LDB1 peaks revealed an enrichment of RUNX1 motifs in both binding site populations, which, however, occurred together with the AP-1 motif in siMM-specific peaks and with C/EBP binding motifs after RUNX1-ETO knockdown (Figure S2G). As expected from it being part of the RUNX1-ETO and RUNX1 complex, LDB1 binding correlated with interactions in control DHSs, which were lost after RUNX1-ETO depletion but also participated in new interactions (Figure S2F, third panel). To test whether LDB1 was required for RUNX1-ETO binding, we depleted it using siRNA knockdown in Kasumi-1 cells with and without RUNX1-ETO knockdown (Figure S3A). LDB1 depletion led to an increase in cell death both by apoptosis and necrosis, but only in RUNX1-ETO-expressing cells, confirming that it is required for the maintenance of the leukemic phenotype (Sun et al., 2013) (Figure S3B). However, LDB1 was not required for RUNX1-ETO binding to chromatin (Figure S3C). LDB1 was also not required for the upregulation or repression of RUNX1-ETO target genes. As expected, RUNX1-ETO knockdown led to increases in expression of the direct RUNX1-ETO target genes *C/EBPA*, *CTSG*, and *NFE2* and decreases in *CD34* and *ERG* expression. Knockdown of LDB1 alone or together with RUNX1-ETO knockdown had no additive or negative effect on RUNX1-ETO target gene expression changes (Figure S3D). We therefore conclude that other factors besides RUNX1-ETO control LDB1 binding and determine its functional impact.

**Figure 3:**
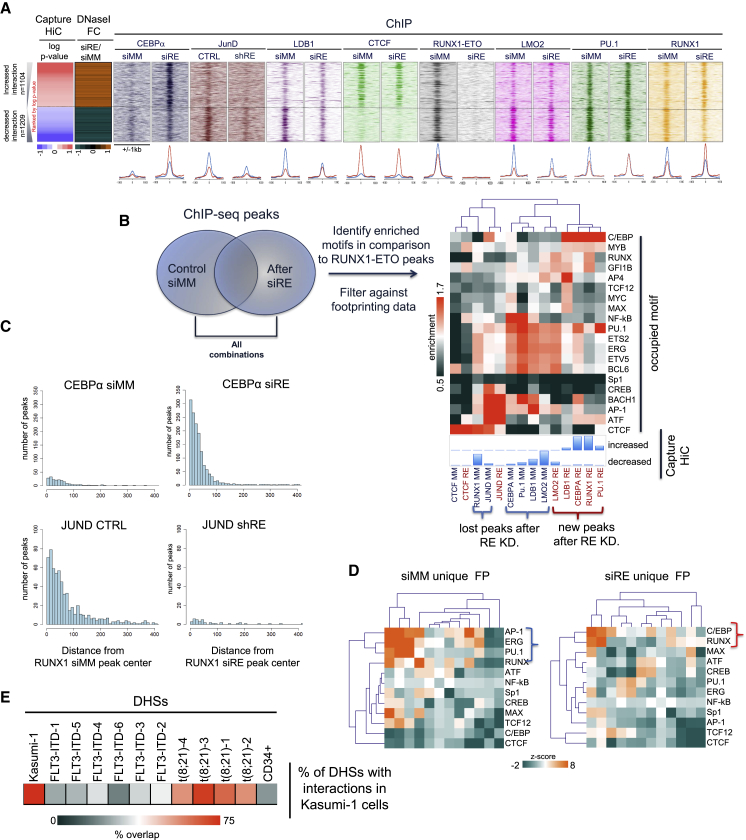
The Cooperation of Constitutive and Inducible TFs Is Associated with Differential Interactions (A) Log p values of the differential interactions were plotted ranked from high to low for control and RUNX1-ETO-depleted cells. Red represents an increase in interaction strength and blue represents a decrease. Alongside, the DNaseI-seq fold difference between control and RUNX1-ETO knockdown cells as well as ChIP-seq density profiles for C/EBPα, JUND, LDB1, CTCF, RUNX1-ETO, LMO2, and PU.1 are plotted from Kasumi-1 cells, transfected with either siMM or siRE as indicated. The panels below show the average profiles of the binding of the indicated TFs plotted around the peak summit for control and RUNX1-ETO-depleted cells. Red, ChIP signal specific for peaks with increased interactions; blue, ChIP signal specific for peaks with decreased interactions. (B) Determination of enriched motifs for other TFs in ChIP-seq peaks specific for control and RUNX1-ETO-depleted cells. Motif enrichment was first identified using HOMER and then filtered against digital footprinting data from day 10 of knockout to ensure that these binding motifs were functional. Enrichment scores were subjected to unsupervised clustering for each of the indicated motifs (on the right). The heatmap depicts the degree of motif enrichment with highly enriched motifs shown in red. Peaks were overlaid with the DHS that show new interactions (red brackets at the bottom) or whose interactions are lost (blue brackets). Enrichment scores were calculated by the level of motif enrichment in the unique peaks, as compared to motif enrichment in RUNX1-ETO peaks. Bottom panels: percentage of peaks showing differential interaction with TFs binding to these sites as determined by ChIP-seq (control cells, blue; RUNX1-ETO-depleted cells, red). (C) Bar plots illustrating the distribution of distances between the binding sites of the indicated TFs as determined by ChIP-seq. We measured the changing distance between RUNX1 peaks in siMM and siRE cells and C/EBPα peaks in siMM (top left) and siRE cells (top right), as well as the distance between RUNX1 peaks and JUND control peaks (bottom left) and JUND after R/E KD (bottom right). (D) Bootstrapping analysis of the significance of co-localizing of footprinted motifs within day-10 DHSs for sites that are either lost (left panel) or gained (right panel) after RUNX1-ETO depletion as compared to the rest of the genome. The heatmap shows the significance of motifs co-localizing within 50 bp as compared to sampling by chance. (E) Heatmap highlighting the percentage of day-4 Kasumi-1 DHSs with interactions found in different patient groups indicating the similarity between cell-line and primary t(8;21) data. The t(8;21) and FLT3-ITD DHS/CHi-C patient data were downloaded from GEO: GSE108316 (Assi et al., 2019).

We next investigated whether the change in interaction was associated with altered TF occupancy. To this end, differential interactions were ranked by fold change in p value (Figure 3A), and associated DHSs were plotted alongside together with C/EBPα, JUND, LDB1, CTCF, RUNX1-ETO, PU.1, and RUNX1 ChIP-seq data. Beneath, we plotted the average profiles of factor binding for control and RUNX1-ETO-depleted cells (blue, ChIP signals associated with lost interactions; red, gained interactions). Differential interactions were associated with the differential binding of some, but not all, TFs. RUNX1-ETO-bound sites were associated with DHSs involved in both decreased and increased interactions, demonstrating that it is not the sole determinant of the interaction pattern. DHS associated with gained interactions showed a strong increase in C/EBPα as well as an increase in RUNX1 binding. Conversely, DHSs associated with decreased interactions after RUNX1-ETO knockdown lost JUND as well as LDB1 binding. An example for a downregulated gene is *CCND2* (Figure S2F), whose expression we have previously shown to be dependent on the presence of AP-1 factors (Martinez-Soria et al., 2019). Increased interactions did not involve an increase in LMO2 or PU.1 binding, and loss of interactions did not involve CTCF*.* The *CITED2* gene is an example of a gene with a new interaction driven by C/EBPα binding (Figure 2G, shaded bar). In summary, our study shows that the main drivers of changes in cis-element interactions are the loss of RUNX1-ETO binding together with the loss of LDB1 and AP-1 binding along with the increased binding of C/EBPα and RUNX1 to new sites.

We next identified additional TFs associated with differential interactions and clustered TF binding motifs enriched in ChIP-seq peaks that either overlapped with new interaction sites or with sites lost after RUNX1-ETO depletion (Figure 3B, left panel and panels below the heatmap). Since the majority of DHS changes participating in differential interactions had already occurred at day 4 of knockdown (Figure 1E), we used our day-10 digital footprinting data to ensure that these motifs were functional and could be occupied. We then calculated the motif enrichment score of such motifs (depicted on the right) (Figure 3B, top-right panel). These analyses showed that the score of enriched motifs for RUNX1 and C/EBP family members increased in differential interactions upregulated after RUNX1-ETO depletion, together with an increase in GFI1, MYB, and MYC/MAX binding site occupancy. In contrast, and in concordance with our ChIP-seq data, AP-1 motif enrichment decreased in interactions that were lost after RUNX1-ETO depletion, together with loss of activating TF (ATF) and nuclear factor κB (NF-κB) motif occupancy. We also detected enrichment of motifs in both gained and lost interactions. This was true for ETS-family factors such as ERG and PU.1, but also for RUNX1, suggesting that factors move to other sites as shown previously (Lichtinger et al., 2012). To confirm this idea, we determined the distribution of distance between RUNX1 binding and other TFs before and after RUNX1-ETO depletion using the ChIP-seq data. This analysis showed a significant co-localization between AP-1 and RUNX1 peaks before, but not after, RUNX1-ETO depletion. In contrast, RUNX1 and C/EBPα show significant co-localization after RUNX1-ETO depletion (Figure 3C). In spite of the appearance of new RUNX1 binding sites after RUNX1-ETO depletion (Ptasinska et al., 2012, Ptasinska et al., 2014), no significant changes were observed in the distribution of distance between RUNX1 and LDB1 and LMO2 and PU.1 peaks (Figures S3F–S3H), indicating no change in this type of factor collaboration. These analyses suggest that RUNX1 cooperates with different factors regulating different biological processes in control and RUNX1-ETO-depleted cells. To examine TF cooperation after the onset of monocytic differentiation, we performed a bootstrapping analysis (Figure 3D) in RUNX1-ETO-depleted and control cells that identified occupied TF binding motifs co-localizing with high significance within 50 base pairs (bp) as compared to the rest of the active genome (highlighted in red). This analysis again confirmed the strong co-association of occupied C/EBP and RUNX1 motifs in differentiated cells and AP-1, ETS, and RUNX motifs co-occurrences in control cells. Interestingly, the AP-1 or C/EBP motifs were not preferentially footprinted in the DHSs shared between control and knockout cells and did not co-localize with other motifs (Figure S3I), indicating that co-localizing (RUNX1-AP-1) sites are part of the AML-specific cistrome. In summary, these analyses demonstrated that the establishment of specific RUNX1-ETO-dependent cis-element interactions are mediated by the cooperation of a limited set of constitutive and inducible TFs. The depletion of RUNX1-ETO drives the loss and relocation of TFs and thus the establishment of new interactions via new factor collaborations.

### The Construction of Transcriptional Networks Grounded in Multi-omics Data

**Figure 4:**
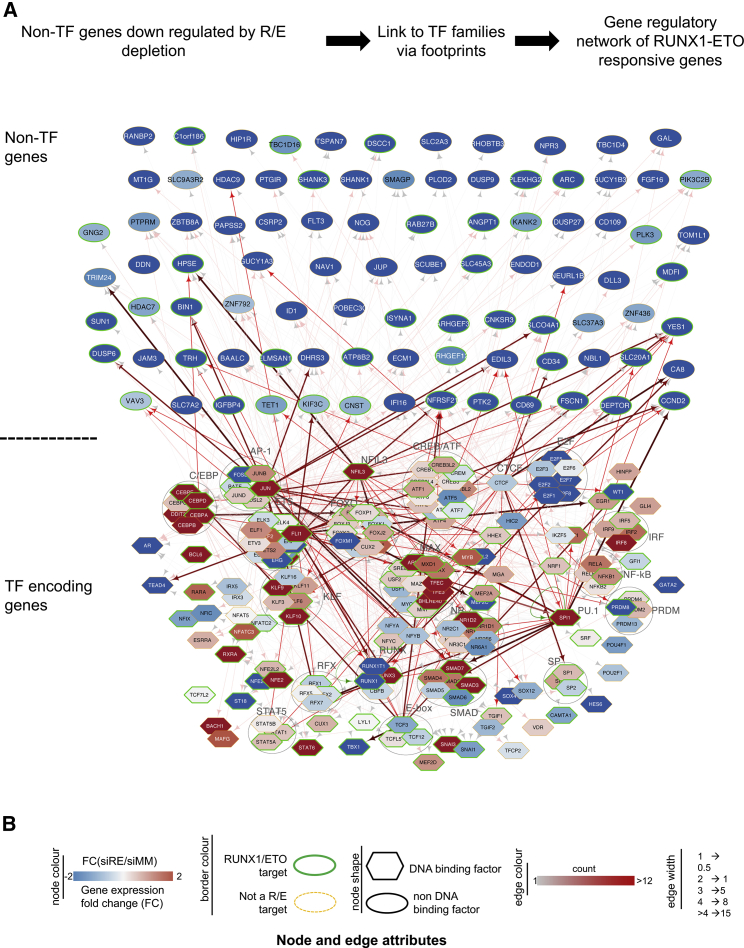
Differentially Expressed Genes after RUNX1-ETO Knockdown Are Regulated by Different TF Networks (A) Top panel: data analysis strategy. Transcriptional network of downregulated (blue) non-TF (effector) genes after RUNX1-ETO knockdown (top rows) connected to genes encoding TF families (bottom rows) as determined by digital footprinting and CHi-C. Arrows going outward can come from any TF family within a group; incoming arrows are specific for each gene. (B) Node and edge attributes.

The Kasumi-1 cell line is one of the best-studied human models of t(8;21) AML with numerous multi-omics data available that should be amenable to modeling approaches predicting transcriptional network behavior in response to perturbation. So far we have assigned factor binding site data only to their nearest promoter. However, numerous studies have shown that such assignments were not accurate (Mifsud et al., 2015, Sanyal et al., 2012). In our study, we found that only about 40% of all cis-regulatory elements in control cells interacted with their nearest promoter. Our CHi-C data enabled us to assign DHSs containing active cis-elements and footprinted regions to promoters (Data S2). More than 70% of all DHSs assigned to their rightful promoter in Kasumi-1 cells were also present in t(8;21) but not in FLT3-ITD patients or normal CD34+ hematopoietic stem cell (HPSCs; Figure 3E; Assi et al., 2019). To construct gene regulatory networks and to examine how these networks shift after RUNX1-ETO depletion and differentiation, we used our footprinting data (control and day-10 siRE) to assign occupied motifs to specific TF families capable of binding to this motif (Table S1, indicated as groups in Figure 4). We then plotted the connections between factors and genes that were downregulated (Figure 4B, blue ovals) or upregulated (Figure S4, red ovals) by at least 2-fold following RUNX1-ETO depletion at day 10, with the former being markers for the leukemic and the latter being markers for differentiated states. We also highlighted which genes were RUNX1-ETO targets (green boundary). This analysis shows a complex web of interactions between effector genes (lined up at the top) and TF encoding genes, many of which are known to respond to RUNX1-ETO depletion, such as *C/EBPA* or *IRF8.* The networks highlight the TFs involved in differentiation, again showing that increased C/EBPα activity is the main driver of the changes of the t(8;21) transcriptional network after RUNX1-ETO depletion, with C/EBP family members binding to multiple differentiation-specific cis-regulatory elements and driving the upregulation of their respective genes (Loke et al., 2018, Ptasinska et al., 2014). An example of a downregulated gene specific to the leukemic state includes *UBASH3B*, which has previously been shown to regulate the proliferation of t(8;21) cells (Goyama et al., 2016). Another such example is *YES1*, which, together with another downregulated gene, *MEIS2*, is involved in maintaining leukemic growth (Vegi et al., 2016). AP-1 members are important for maintaining the leukemic growth phenotype, as shown by expressing a dominant-negative FOS protein in t(8;21) cells. Expression of this peptide downregulates the expression of several cell cycle genes, including *CCND2* (Martinez-Soria et al., 2019), and blocks tumor growth *in vivo* (Assi et al., 2019). Such examples of properly annotated RUNX1-ETO-responsive genes with known function show the quality of our analysis with respect to the prediction of important genes required for AML maintenance. Last but not least, our studies serve as paradigm for how high quality multi-omics data can be used to generate in-depth information on the regulatory circuitries of a specific type of AML.

## STAR★Methods

### Key Resources Table

**Table:** 

REAGENT or RESOURCE	SOURCE	IDENTIFIER
**Antibodies**		

ETO	Diagenode	Cat# C15310197
RUNX1 (C-terminal epitope)	Abcam	Cat# 23980; RRID: AB_2184205
C/EBPα	Abcam	Cat# 40761; RRID: AB_726792
LDB1	Abcam	Cat# 96799; RRID: AB_10679400
LMO2	R&D	Cat# AF2726; RRID: AB_2249968
CTCF	Abcam	Cat# 70303; RRID: AB_1209546
JUND	Santa Cruz	Cat# sc74; RRID: AB_2130177
GAPDH	Sigma	Cat# F3165; RRID: AB_259529
Anti-Rabbit HRP	Cell signalling	Cat# 7074; RRID: AB_2099233
Anti-Mouse HRP	Jackson	Cat# 115-0350-62; RRID: AB_2338504
CD34 Monoclonal Antibody (4H11), PE	eBioscience	Cat# 12-0349-42; RRID: AB_1548680
c-Kit Monoclonal Antibody (104D2), FITC	eBioscience	Cat# 11-1178-42; RRID: AB_2572472

**Oligonucleotides**		

Oligonucleotide sequences, see Table S2	This Paper	NA

**Chemicals, Peptides, and Recombinant Proteins**		

TruSeq Stranded mRNA with Ribo-Zero human assay	Illumina	Cat# 20020596
TruSeq RNA Sample Prep kit	Illumina	Cat#RS-122-2001
KAPA hyper Prep Kit	Kapa Biosystems	KK8500
KAPA Library Quantification kit	Kapa Biosystems	Cat#KK4824
MinElute Gel Extraction Kit	QIAGEN	Cat#28604
DNaseI	Worthington,	DPPF grade
AMPure XP beads	Beckman Coulter	Cat#A63882
NextSeq500 High output 150 cycles	Illumina	Cat#FC-404-2002
NextSeq500 High output 75 cycles	Illumina	Cat#FC-404-2005
Nucleospin RNA column	Machery Nagel,	740955.50
OligoDT primer	Promega	C110A
Murine Moloney Virus reverse transcriptase	Promega	M170A
RNase Inhibitor	Promega	N261A
Sybr Green mix	Applied Biosystems	4309155
Proteinase K	Roche	03115801001
Di(N-succinimidyl) glutarate (DSG)	Sigma-Aldrich	80424
MyOne Streptavidin C1	DynaBeads Invitrogen	65601
MyOne Streptavidin T1	DynaBeads Invitrogen	65001
Formaldehyde	(Pierce, Thermos Scientific, USA	28906
(NEB)2 buffer	NEB	37002

**Critical Commercial Assays**		

Dead Cell Removal microbeads	Miltenyi Biotec	130090101
Annexin V-APC/PI staining	Ebiosciences	88-8007-74
High Sensitivity DNA Chips	Agilent Technologies	5067-4626
RNA Pico Chips	Agilent Technologies	5067-1513
High Pure PCR Product Purification Kit	Roche	11732676001
SureSelect target enrichment	Agilent Technologies	5190-4393
HindIII	NEB	R0104M

**Deposited Data**		

CHIP-seq data	This study	GSE121282
DNaseI-seq data	This study	GSE121282
RNA-seq data	This study	GSE121282
Capture HiC	This study	GSE117108
reprogramming ATAC-Seq	Corces et al., 2016	GSE75384
Published ChIP-Seq	Ptasinska et al., 2014	GSE60131

**Experimental Models: Cell Lines**		

Kasumi-1 human cell line	DSMZ	ACC220

**Software and Algorithms**		

Bowtie 2	Langmead and Salzberg, 2012	http://bowtie-bio.sourceforge.net/bowtie2/index.shtml
MACS2	Zhang et al., 2008	https://github.com/taoliu/MACS
Homer	Heinz et al., 2010	http://homer.ucsd.edu/homer/motif/
Cufflinks v2.2.1	Trapnell et al., 2013	http://cole-trapnell-lab.github.io/cufflinks/announcements/protocol-paper/
Bedtools	Quinlan and Hall, 2010	https://bedtools.readthedocs.io/en/latest/
STAR	Dobin et al., 2013	https://github.com/alexdobin/STAR
Wellington algorithm	Piper et al., 2013	https://pythonhosted.org/pyDNase/
Illustrator	Adobe System Software Ireland	https://www.adobe.com/cn/products/cs6/illustrator.html
ZEN	Zeiss	https://www.zeiss.com/microscopy/int/microscope-cameras.html
GraphPad Prism 6.0	GraphPad Software	https://www.graphpad.com/scientificsoftware/prism/

### Lead Contact and Materials Availability

Further information and requests for resources and reagents should be directed to and will be fulfilled by the Lead Contact, Constanze Bonifer (c.bonifer@bham.ac.uk).

This study did not generate new reagents.

### Experimental Model and Subject Details

#### Cell Line Culture

Cells were maintained in a humidified incubator at 37°C with 5% CO2. t(8;21) Kasumi-1 cells were cultured in RPMI with 10% FCS supplemented with 1% glutamine and 1% penicillin/streptomycin.

### Method Details

#### siRNA Mediated Depletion of RUNX1-ETO or LDB1

1x10^7^ cells were electroporated using a EPI 3500 (Fischer, Germany) single 350 V pulse for 10ms. After electroporation, the cells remained in their cuvettes for 10 minutes before being directly added to RPMI-1640 with 10% FCS, supplemented with penicillin/streptomycin and glutamine at a concentration of 0.5 x10^6^ cells per ml and returned to an incubator kept at 37°C and 5% CO_2_. siRNA sequences (SIGMA ALDRICH Germany) specific for the translocation breakpoint of RUNX1-ETO were 5′ CCUCGAAAUCGUACUGAGAAG −3′ (sense) and 5′- UCUCAGUACGAUUUCGAGGUU-3′ (antisense). Control siRNA was 5′-CCUCGAAUUCGUUCUGAGAAG-3′ (sense) with 5′-UCUCAGAACGAAUUCGAGGUU-3′ (antisense). siRNA sequences specific for LDB1 ON-TARGETplus Human LDB1 siRNA SMARTpool (L-016010-00-0005, Dharmacon). siRNA was used at 200 nM.

#### RNA Extraction

RNA from Kasumi-1 cells was purified using a Nucleospin RNA column (Machery Nagel, France), according to manufacturer’s instructions. The quality of RNA from was assessed using a spectrophotometer, by the ratio of the absorbance at 260 nM and 280 nM wavelengths. RNA has a greater absorbance in the 260 nM wavelength, Eukaryotic Total RNA PICO Bioanalyser chip (Agilent technologies, USA) allows visualization of the size of the RNA molecules and thus, demonstrates whether the sample is degraded or not.

#### RNA Seq Libraries

RNA-seq libraries were prepared with a Total RNA Ribo-zero library preparation kit (with ribosomal RNA depletion) (Illumina, USA) according to manufacturer’s instructions with the following alterations: 15 cycles of PCR was undertaken to amplify the library and adaptors for multiplexing were used at a 1:4 dilution. Library quality was checked by running the samples on a Bioanalyser and libraries were quantified using a Kapa library quantification kit (Kapa Biosystems, USA) and run in a pool of eight indexed libraries in two lane of a HiSeq 2500 (Illumina, USA) using rapid run chemistry with 100bp paired end reads.

#### cDNA Synthesis

1 μg RNA was used to make cDNA with 0.5 μg OligoDT primer, Murine Moloney Virus reverse transcriptase and RNase Inhibitor (Promega, USA) according to the manufacturer’s protocol.

#### Real-Time Polymerase Chain Reaction

RT-PCR was performed using Sybr Green mix (Applied Biosystems, UK), at 2x dilution. Primers were used at 100 nM final concentration. cDNA was diluted 1:50 depending on expression levels of targets. A 7900HT system (Applied Biosystems, UK) was used to perform qPCR. Primers used in this project are listed in Table S2.

#### Dead Cell Removal and Annexin V/PI Staining for Flow Cytometry

Dead cell removal was performed using negative selection on a MS column following incubation with Dead Cell Removal microbeads (Mitenyi Biotech, USA) as per manufacturer’s instructions. Dead cell removal was performed on all samples prior to RNA extraction or DHSs mapping. Annexin V-APC/PI staining (Ebiosciences, USA) or was performed according to manufacturer’s instructions.

#### DNaseI Hypersensitivity Site Mapping

Prior to DNaseI digestion, apoptotic cells were removed using the Dead Cell Removal Kit (Miltenyl Biotech, UK) as per manufacturer’s instructions. 3x 10^7^ Kasumi-1 cells were suspended in 1 mL DNase I buffer (0.3M sucrose, 60 mM KCl, 15 mM NaCl, 5 mM MgCl2, 10 mM Tris pH7.4). Digestion on 4.5x10^6^ cells was performed with DNase I (Worthington, DPPF grade) at 80 units/ml in DNase I buffer with 0.4% NP-40 and 2 mM CaCl2 at 22°C for 3 minutes. The reaction was stopped with cell lysis buffer (0.3M NaAcetate, 10mM EDTA pH 7.4, 1% SDS) with 1mg/ml Proteinase K and incubated at 45°C overnight. The digested DNase I material was treated with RNase A (Sigma Aldrich, Germany) at a final concentration of 100 μg/ml at 37°C for 1 hr. Genomic DNA was extracted using phenol/chloroform method: an equal volume of phenol was added to the reaction and placed on a rotator wheel for 45 minutes. This was centrifuged for 5 minutes at 16000 x g at room temperature. The top layer was transferred to a new tube and the process was repeated sequentially with phenol/chloroform and chloroform. After purification by chloroform extraction, genomic DNA was precipitated with ethanol. This was pelleted by centrifugation for 5 minutes, at 16000 x g at 4°C. The pellet was resuspended with 70% ethanol and centrifugation for 5 minutes, at 16000 x g at 4°C. The pellet was air-dried and dissolved by Tris-EDTA (40 mM Tris Acetate 1 mM EDTA). Digestion was checked visually by running the samples on a 0.7% agarose gel and by RT-PCR evaluating the ratio of open (TBP promoter) to closed regions of DNA (chromosome 18) and active gene body (beta-actin) to prevent selection of over digested samples. Primers used in this project are listed in Table S2. Subsequently, between 2 to 10 μg of DNase I-digested DNA (depending on material available) were run on a 1.5% agarose gel for selection of shorter fragments to increase the fraction of fragments captured from DHSs. Prior to loading on gel, the purified DNA was treated again with RNase A (Sigma Aldrich, USA) at a final concentration of 100 μg/ml at 37°C for 1 hr. 50-300 bp fragments were isolated and purified from the gel using a MinElute gel extraction kit (QIAGEN, USA) as per manufacturer’s instructions and validated by qPCR. Following this, the size selected sample was validated again by RT-PCR, this time using shorter amplicons to enable detection of the shorter fragments enriched by the size selection process.

#### Library Production of DNase I Material for High Throughput Sequencing

After size selection, a library was prepared using KAPA Hyper Prep Kit sample preparation kit (Kapa Biosystems, USA) as per manufacturer’s protocol. After PCR a final size selection step was performed by running the library on 2% TAE gel, followed by excision of 190-250 bp sized gel fragment. The library was purified from the gel using a MinElute gel extraction kit (QIAGEN, USA). The quality of the libraries was assessed on an Agilent 2100 Bioanalyser. Libraries were subsequently run on two lanes of an Illumina HiSeq 2500 flow-cell for transcription factor footprinting, or as part of 12 indexed libraries in one lane of a NextSeq500 (Illumina, USA) for DHS mapping alone.

#### ChIP-qPCR and ChIP-Seq Library Preparation

##### Double Cross-Linking

A double cross-linking technique was used to optimize the efficiency of transcription factor chromatin immunoprecipitation (ChIP). 2x10^7^ cells were washed thrice in PBS. Di(N-succinimidyl) glutarate (DSG) (Sigma-Aldrich, Germany) at 850 μg/ml was added to 2x10^7^ cells per ml and were incubated for forty-five minutes. Cells were washed four times and fixed with 1% formaldehyde (Pierce, Thermos Scientific, USA) for ten minutes. Glycine to produce a final concentration of 100mM was added to stop the reaction. The pellet was washed again with PBS. Buffer A (HEPES pH 7.9 10 mM, EDTA 10 mM, EGTA 0.5 mM, Triton x100 0.25%, complete mini protease inhibitor cocktail (PIC) 1x (Sigma-Aldrich, Germany) was added for 10 mins at 4°C and removed by centrifugation at 500 g for 5 minutes. This was repeated with buffer B (HEPES pH 7.9 10 mM, EDTA 1 mM, EGTA 0.5 mM, Triton x100 0.01%, PIC 1x). The residual nuclei were then spun down at 16000 x g at 4°C for 5 minutes and aliquoted at 2x10^7^ cells for 4 immunoprecipitations.

##### Chromatin Immunoprecipitation (ChIP)

Each aliquot of 2x10^7^ cells was re-suspended in 600 μL of sonication buffer (Tris-HCL pH 8 25 mM, NaCL 150 mM, EDTA 2 mM, Triton 100x 1%, SDS 0.25%, Protease inhibitor cocktail (PIC) 1x). 300 μL of nuclei in sonication buffer was placed in each polystyrene tube and sonicated at 75% amplitude, 26 cycles: 30 s on and 30 s off per cycle (Q800, Active Motif, USA). Subsequently, 1.2ml of dilution buffer (Tris-HCL pH8 25 mM, NaCL 150 mM, EDTA 2 mM, Triton 100x 1%, glycerol 7.5%, PIC 1x) was added to the pooled post sonication material. This was divided equally between four immunoprecipitations (with 5% of input taken for validation). 20 μL protein G beads (Diagenode, Belgium) were washed twice with 500 μL of 50 mM citrate phosphate buffer and once with 100 mM sodium phosphate. 4 μg antibody ETO (Santa Cruz) or 4 μg antibody AML1-ETO (15310197, Diagenode), or RUNX1 (Ab23980, Abcam) or 4μg antibody C/EBPα (A2814, Santa Cruz) or 2μg antibody LBD1 (96799, Abcam) or 2μg antibody LMO2 (AF2726, R&D) or 2μg antibody CTCF (70303, Abcam) or 2μg JUND (sc74, Santa Cruz) was added to 10 μL 100 mM sodium phosphate, 0.5% BSA and incubated with protein G beads at 4°C for 1 hour. Chromatin was then added to the protein G beads with antibody and returned to 4°C for 4 hours. Unbound chromatin was separated from the beads by magnet and the attached beads were washed by buffer 1 (Tris HCL 20 mM, NaCl 150 mM, EDTA 2 mM, Triton x100 1%, SDS 0.1%), twice with buffer 2 (Tris HCL 20 mM, NaCl 500 mM, EDTA 2 mM, Triton x100 1%, SDS 0.1%), LiCL buffer (Tris HCL 10 mM, LiCl 250 mM, EDTA 1 mM, NP40 0.5%, sodium deoxycholate 0.5%) and finally twice with wash buffer 4 (Tris HCL pH8, 10 mM, NaCl 50 mM, EDTA 1mM). The column was eluted twice with 50 μL buffer (NaHCO_3_ 100 mM and SDS 1%) and the eluant containing the chromatin was pooled. Crosslinks were reversed by incubating the samples at 65°C overnight in 500 mM NaCl, 500 μg/ml proteinase K. DNA was purified by Ampure beads (Beckman Coulter, USA), as above, with the DNA eluted with 50 μL water. Validation of the ChIP was performed by qPCR using a standard curve of genomic DNA from untreated Kasumi-1 cells (10ng/ μl followed by serial 1:5 dilutions). The input material was diluted 1:5 with water. Primers used in this project are listed in Table S2. Validation was analyzed as a ratio of the qPCR signal from the ChIP material over the input.

##### Library Production of ChIP Material for High Throughput Sequencing

Libraries for high throughput sequencing were prepared using the Tru-seq DNA sample preparation kit (Illumina, USA) or Kapa HyperPrep kit (Kapa Biosystems, USA), as per manufacturer’s protocol. 18 cycles of PCR was performed and 200-350bp fragments were size selected by running the samples in an agarose gel. Libraries were purified from the gel using a MinElute Gel extraction kit (QIAGEN, USA). Libraries were validated by qPCR, with an analysis of the ChIP signal of a positive control region (e.g., PU.1 3H enhancer) over a negative control region (e.g., *IVL*). Finally, libraries were quantified by Kapa library quantification kit (Kapa Biosystems, USA) and run in a pool of four indexed libraries in one lane of a HiSeq 2500 (Illumina, USA) or 12 indexed libraries in one lane of a NextSeq 500 (Illumina, USA) using 50 cycle single-end reads.

##### Circularized Chromosome Conformation Capture (4C-seq)

4C analysis was performed exactly as described in Gröschel et al. (2014). 1x10^7^ Kasumi-1 cells, transfected with mismatch siRNA (siMM) or siRNA specific to siRUNX1-ETO (siRNA), were fixed with 2% formaldehyde and incubated for 10 minutes at room temperature. 1.425 mL of 1M glycine was added to quench the cross-linking reaction. Fixed cells were immediately centrifuged for 8 minutes at 4°C, 500 xg. Supernatant was removed and the pellet resuspended in 1ml lysis buffer (500μl 1M TRIS pH 7.5, 300μl 5M NaCl, 100μl 0.5M EDTA, 250μl 20% NP-40 and 100μl Triton X-100 made up to 10ml with H2O) and incubated at room temperature for 5 minutes, followed by 5 minutes at 65°C. Cells were then kept on ice while complete cell lysis was determined via Trypan blue (GIBCO) staining. Cells were centrifuged at 800 xg for 5 minutes and the pellet was taken up in 440 μl H20 and 60 μl 10X RE buffer 2 (NEB). 15 μl of SDS was added and the tube placed at 37°C for 1 hour. 75 μl of 20% Triton X-100 was added and the tube incubated at 37°C for 1 hour. A 5 μl aliquot was removed as an ‘undigested control’ sample before 200 units of the restriction enzyme DpnII was added. The tube was incubated for 4 hours at 37°C, and then another 200 units of DpnII was added, followed by an overnight 37°C incubation. The following day 200 units of DpnII was added for 4 hr at 37°C. A 5 μl aliquot was removed as a ‘digested control’ sample. To this, along with the ‘undigested’ sample, 90 μl of 10mM Tris pH 7.5 and 5μl Proteinase K (10 mg/ml) was added to reverse the cross links. These control samples were run on a 0.6% agarose gel to assess the digestion efficiency. All 37°C incubations were conducted in a heated block, shaking at 900 RPM. DpnII was selected as the restriction enzyme as it functions in SDS, and combined with the second restriction enzyme (Csp6I) it generates restriction fragments near the target loci, with a suitable size for efficient ligation and PCR amplification. Both of these enzymes are 4bp cutters, so will cut the genome into 256 bp fragments, on average. This allows for a high resolution assay. The DpnII was inactivated by incubation at 65°C for 20 minutes. On ice, 700 μl of 10X ligation buffer, 7 mL of milli-Q H20 and 10 μl T4 Ligase (Roche 5U/μl) were added then samples were incubated overnight at 16°C. The following day, to assess ligation efficiency, a 100 μl aliquot of the sample was taken as the ‘ligated control’. The crosslinks were reversed as above and the sample run on a 0.6% agarose gel. To reverse the crosslinks, 30 μl Prot K (10mg/ml) was added and samples were left overnight at 65°C. The next day, 30 μl RNase A (10mg/ml) was added and samples were incubated for 45 minutes at 37°C. DNA was extracted by adding 7 mL phenol-chloroform. Samples were mixed thoroughly then centrifuged at 3000 xg at room temperature. The water phase was transferred to a new 50 mL tube to which 7 mL of milli-Q H20, 7 μl of glycogen, 1.5 mL 2M NaAC pH 5.7 and 35 mL ethanol was added. Samples were placed at –80°C overnight. The next day samples were centrifuged at 4°C for 30 min, 3000 xg. The supernatant was removed and 10 mL of cold 70% ethanol was added. Samples were centrifuged again for 15 min, 3000 xg at 4°C. The supernatant was removed and the pellet left to dry at room temperature. The pellet was dissolved in 150μl 10mM Tris pH 7.5. Each sample was transferred to a 1.7 mL tube, 50 μl 10X restriction buffer and 50 units of the restriction enzyme Csp6I (Fermentas # ER0211) was added and the volume made up to 500 μl with milli-Q H20. After an overnight incubation, 500 RPM shaking, at 37°C, a 5 μl aliquot of the sample was taken. This ‘digestion control’ was run on a 0.6% agarose gel. The enzyme was inactivated as previously describe and the samples transferred to a 50 mL tube. 1.4 mL of 10X ligation buffer and 20 μl of ligase (100 U) (Roche Catalog # 10799009001) was added, then the reaction made up to 14ml with milli-Q H2O. After an overnight ligation at 16°C, 1.4ml 2M NaAC pH 5.6, 14μl glycogen and 35ml of 100% ethanol were added. Samples were stored at –80°C overnight. The next day samples were centrifuged at 4°C for 45 minutes, at 3750 RPM. The supernatant was removed and 15 mL of cold 70% ethanol was added. The samples were then centrifuged again for 15 minutes, at 20°C and 3750 RPM. Again, the supernatant was removed and the pellet then left to dry at room temperature. Once dry the pellet was dissolved in 150 μl 10mM Tris pH 7.5 at 37°C. Samples were then purified using a QIAquick PCR purification kit, according to the manufacturer’s protocol. Samples were eluted in 50 μl 10mM Tris pH 7.5 and pool samples. DNA concentration of each 4C template was determined via analysis with a NanoDrop 2000 (Thermo Scientific). Restriction fragments greater than 350 bp and within 2kb of the target genomic region were selected as viewpoint fragments, dependent on the ability to design specific primers. A 5′ Illumina adaptor sequence was added so the inverse-PCR products did not need further processing prior to sequencing. Reading primers were designed as close to the primary restriction site as possible, to reduce reads from the known viewpoint sequence. Non-reading primers were designed to regions less than 120kb from the secondary restriction site. 200 ng of 4C template was used per PCR reaction. For each viewpoint and template, 16 PCR reactions were conducted using an Expand Long Template system (ROCHE # 11681834001) (see Table S2 for primer sequences). The pooled PCR products (total volume 800 μl) were then purified using the High Pure PCR Product Purification Kit (Roche cat. no. 11732676001), to remove any adaptor containing primers (< 120 bp). Samples were centrifuged to pellet any beads that escaped the column. The supernatant was taken, then the concentration and purity of this 4C template was assessed by a NanoDrop 2000 (Thermo Scientific) (260/280 ratio >2 and 260/230 ratio >1.8 was required). The libraries were then visualized on a 1.5% agarose gel. All 4 of the 4C libraries were pooled, and then multiplexed sequencing was performed on the HiSeq 2500 platform. Individual fragment counts were calculated for every 1kb bin. A median was calculated, with a 3kb sliding window, and data from both biological replicates was merged. The R package DESeq2 was used to calculate the log2 fold change (RUNX1/ETO knockdown versus control) at the local genomic coordinates. Viewpoint specific 4C-seq PCR primers used in this project are listed in Table S2.

##### Hi-C Library Generation

Hi-C library generation was carried out as described previously (Mifsud et al., 2015, Lieberman-Aiden et al., 2009), with the following modifications which were detailed with the following modifications. After fixation in 2% formaldehyde for 5 min, 50 million Kasumi-1 cells were homogenized in 10 mL of ice-cold lysis buffer ten times on ice with a tight pestle, incubated on ice for 15 min and then homogenized a further ten times. After overnight digestion with HindIII at 37°C, DNA ends were labeled with biotin-14–dATP (Life Technologies) in a Klenow end-filling reaction. After phenol-chloroform purification, the DNA concentration was measured using Quant-iT PicoGreen (Life Technologies), and 40 μg of DNA was sheared to an average size of 400 bp, using the manufacturer’s instructions (Covaris). The sheared DNA was end repaired, adenine tailed, and double size selected using AMPure XP beads to isolate DNA ranging from 250 to 550 bp in size. Ligation fragments marked by biotin were immobilized using MyOne Streptavidin C1 DynaBeads (Invitrogen) and ligated to paired-end adaptors (Illumina). The immobilized Hi-C libraries were amplified using PE PCR 1.0 and PE PCR 2.0 primers (Illumina) with 8 PCR amplification cycles.

##### Biotinylated RNA Bait Library Design

Biotinylated 120-mer RNA baits were designed to target both ends of HindIII restriction fragments that overlap Ensembl promoters of protein-coding, noncoding, antisense, snRNA, miRNA and snoRNA transcripts. A target sequence was valid if its GC content ranged between 25 and 65% and the sequence contained no more than two consecutive Ns and was within 330 bp of the HindIII restriction fragment terminus.

##### Promoter Capture Hi-C

Capture HiC of promoters was carried out with SureSelect target enrichment, using the custom-designed biotinylated RNA bait library and custom paired-end blockers according to the manufacturer’s instructions (Agilent Technologies). After library enrichment, a post-capture PCR amplification step was carried out using PE PCR 1.0 and PE PCR 2.0 primers with 4 PCR amplification cycles. CHi-C libraries were sequenced on the Illumina HiSeq 1000 platform.

##### Western Blotting

Protein extracts were prepared using a co-immunoprecipitation kit (Active Motif, USA). Protein extracts were quantified using Bradford protein reagent (Bio-Rad, USA) and 595nM absorbance quantified by spectrophotometry. Absolute concentrations were determined using a standard curve from a known concentration of BSA (Pierce, USA). Protein extracts was run on an acrylamide gel and transferred to nitrocellulose membrane. The antibodies used in this project are listed in Table S2. Enhanced chemiluminescence by SuperSignal PICO (Thermos Scientific, USA) was used to develop the membrane. Chemiluminescence was detected using either developer or Chemidoc XRS system (BioRad, USA).

##### Antibody Staining for Flow Cytometry

15x10^4^ were centrifuged at 300xg and washed with MACS buffer. The cell pellet was re-suspended in 50 μl MACS buffer and 2 μl of antibody was added and incubated for 15 minutes at 4°C in the dark. After incubation, the cells was washed once with MACS buffer before resuspension in 300 μl MACS buffer and analyzed on Cyan ADP (Beckman Coulter, USA). Data were analyzed on Summit 4.3 (Beckman Coulter, USA). Antibodies used in this project are listed below. CD34 Monoclonal Antibody (4H11), PE, eBioscience Cat #12-0349-42; CD117 (c-Kit) Monoclonal Antibody (104D2), FITC, eBioscience Cat #11-1178-42

### Quantification and Statistical Analysis

#### DNaseI Sequencing Data Analysis

DNaseI sequences from all experiments were mapped onto the reference human genome (hg38), with Bowtie version 2.3.1 (Langmead and Salzberg, 2012) using default parameters. Low quality reads were trimmed using Trimmomatic-0.36 (Bolger et al., 2014) and quality control (QC) statistics were obtained using FastQC tools. Unique aligned reads were used for downstream analysis. DNaseI Hypersensitive Sites (DHSs) were called with MACS2 using callpeak function (nomodel, call-summits and q = 0.005 parameters) (Zhang et al., 2008). Clustering of DNaseI-seq samples was carried out using the merged DHSs. The number of reads that mapped to these DHSs was counted in a 400bp window centered on the DHS summit, and subsequently normalized to total sample size using DEseq2 (Love et al., 2014). Pearson correlation coefficients were then calculated for each pair of samples using the log2 of the normalized read counts, and then hierarchically clustered using Euclidean distance and complete linkage clustering of the correlation matrix in R.

#### ChIP Sequencing Data Analysis

ChIP sequencing reads were aligned to the human genome version hg38 with Bowtie version 2.3.1 (Langmead and Salzberg, 2012). Reads that mapped uniquely to the genome were retained and duplicated reads were removed using the MarkDuplicates function in Picard tools (http://broadinstitute.github.io/picard/). Peaks were identified with MACS version 1.4.2 (Zhang et al., 2008) and DFilter software (Kumar et al., 2013) with recommended parameters (-bs = 100 -ks = 50 –refine). Peaks common to both peak calling methods were considered for further analysis.

#### Average Tag Density Profile and Heatmap

The tag density and average profiles for Figures S1D and S2E were generated by calculating the tag density normalized as coverage per million within 400bp windows of the DNaseI peak summit. The read counts for all union peaks were computed. Coverages were calculated for all union peaks and ranked by log2 fold change. Heatmap images were generated via Java TreeView (http://jtreeview.sourceforge.net/) and average profiles were plotted using R.

#### RNA-Seq Data Analysis

RNA-seq reads downloaded from GSE54478 were aligned to the human genome hg38 build with STAR (Dobin et al., 2013) using ENCODE recommend parameters. Separate density profiles for the positive and negative strand were generated using bedtools. Cufflinks (Trapnell et al., 2013) was used to calculate the expression values as Fragments Per Kilobase per Million aligned reads (FPKM) from the aligned RNA-seq data and differentially expressed genes were extracted using the limma R package (Ritchie et al., 2015). All genes with p value ≤ 0.01 were considered with at least 2-fold changes between before and after RUNX1-ETO knock down.

#### Promoter Capture HiC Data Analysis

The CHi-C paired-end sequencing reads were aligned to the human genome hg38 build using HiCUP pipeline (Wingett et al., 2015). The raw sequencing reads were initially separated and mapped against the reference genome. The reads were then filtered for experimental artifacts and duplicate reads, and then re-paired. Statistically significant interactions were called using GOTHiC package (Mifsud et al., 2017) and HOMER software (Heinz et al., 2010). This uses a cumulative binomial test to detect interactions between distal genomic loci that have significantly more reads than expected by chance, by using a background model of random interactions. This analysis assigns each interaction with a p value, which represents its significance. Differential interactions between control and after RUNX1-ETO knock-down were determined with HOMER (Heinz et al., 2010), p value cutoff with at least 0.01 was considered.

#### 4C-Seq Data Analysis

4C-seq data analysis was performed using 4Cseqpipe, as described in van de Werken et al. (2012). Sequence extraction, mapping, normalization, and plotting of cis-contact profiles around PU.1 promoter and enhancer viewpoints were done using packages called by 4Cseqpipe tools. Custom restriction site tracks were built using the -build_re_db option of 4Cseqpipe for the hg19 human genomic version with HindIII as first and second restriction cutters. 4C reads were mapped to the custom hg19 tracks with the in-built 4Cseqpipe mapper. Near-*cis* domainograms were generated for PU.1 viewpoints using the median stat type and plotting the 20th and 80th quantile of the distribution of normalized contact intensities for 5kb sliding windows.

#### Motif Identification and Clustering

De novo motif analysis was performed on peaks using HOMER (Heinz et al., 2010). The annotatePeaks function in HOMER was used to find occurrences of motifs in peaks. In this case we used known motif position weight matrices (PWM) from HOMER database.

Motif clustering: Digital footprinting of DNaseI high-depth sequencing data was performed using the Wellington algorithm (Piper et al., 2013) with FDR = 0.01. For the heatmap that shows hierarchical clustering of motif occurrences within specific and common footprints (Figure 3D). The distance between the centers of each motif pairs was calculated and the motif frequency was counted if the first motif was within 50bps distance from the second motif. Z-scores were calculated from the mean and standard deviation of motif frequencies observed in random sets using bootstrapping analysis. For bootstrapping, peak sets with a population equal to that of the footprinted peaks were randomly obtained from the union of DNase-Seq footprints.

### Data and Code Availability

The CHIP-seq data, DNaseI-seq data, and RNA-seq data generated during this study are available at GEO: GSE121282. The Capture HiC data generated during this study are available at GEO: GSE117108. The published article includes reprogramming ATAC-Seq (Corces et al., 2016) GEO: GSE75384 analyzed during this study. The published article includes Published ChIP-Seq (Ptasinska et al., 2014) GEO: GSE60131 analyzed during this study.

## Supplemental Information

Document S1. Figures S1–S4 and Tables S1 and S2

Data S1. Expression Values of Differentially Expressed Genes during a Time Course of RUNX1-ETO Knockdown

Data S2. Annotation of DHSs to Their Rightful Promoters

Document S2. Article plus Supplemental Information
