# RUNX1-ETO Depletion in t(8;21) AML Leads to C/EBPα- and AP-1-Mediated Alterations in Enhancer-Promoter Interaction

**DOI:** 10.1016/j.celrep.2019.10.075

**Published:** 2019-11-12

**Authors:** Anetta Ptasinska, Anna Pickin, Salam A. Assi, Paulynn Suyin Chin, Luke Ames, Roberto Avellino, Stephan Gröschel, Ruud Delwel, Peter N. Cockerill, Cameron S. Osborne, Constanze Bonifer

(Cell Reports *28*, 3022–3031.e1–e7; September 17, 2019)

In this article, the name of author Stefan Gröschel was incorrectly written as Stephan Gröschel. The article has now been corrected online. The authors regret the error.

