# Supplementary material for: RUNX1-ETO Depletion in t(8;21) AML Leads to C/EBPα- and AP-1-Mediated Alterations in Enhancer-Promoter Interaction
Source: Cell Rep. 2019 Sep 17;28(12):3022–3031.e7. doi: 10.1016/j.celrep.2019.08.040 (PMC6899442; doi:10.1016/j.celrep.2019.08.040)
Supplement: Document S1. Figures S1–S4 and Tables S1 and S2 [file mmc1.pdf]

Cell Reports, Volume *28*

Supplemental Information

RUNX1-ETO Depletion in t(8;21) AML Leads to C/EBPα- and AP-1-Mediated Alterations in Enhancer-Promoter Interaction

Anetta Ptasinska, Anna Pickin, Salam A. Assi, Paulynn Suyin Chin, Luke Ames, Roberto Avellino, Stefan Gröschel, Ruud Delwel, Peter N. Cockerill, Cameron S. Osborne, and Constanze Bonifer

Figure 1 supplementary

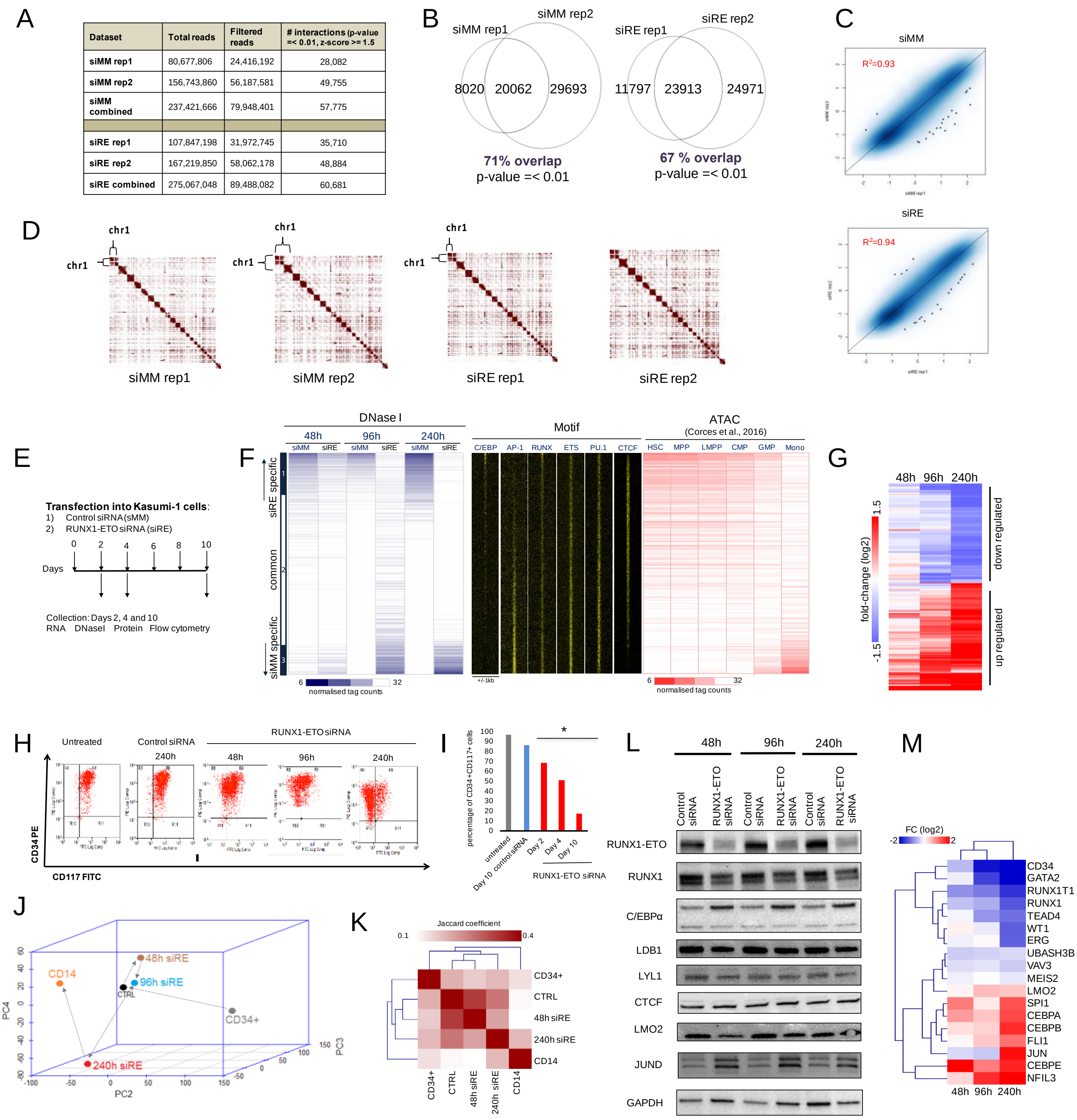

Supplemental Figure 1 (refers to Figure 1)

RUNX1-ETO depletion changes local cis-regulatory element interactions

A: The number of high confidence reads associated within open chromatin regions for two independent Capture HiC experiments from control (siMM) and RUNX1-ETO depleted (siRE) cells and.

B: 2-way Venn diagram showing the overlap of interactions between Capture HiC replicates.

C: Smooth scatter plot showing the correlation between capture HiC replicates based on first principle component analysis.

D: Contact matrix across the whole genome. Each pixel represents a 10 Mb section of the genome. Colour intensity represents the interaction frequency. Left hand plots show the interaction matrices generated with data from Kasumi-1 cells transfected with siMM for four days (biological replicate 1 and 2). Right hand plots show the interaction matrices generated with data from Kasumi-1 cells transfected with siRE for four days (biological replicate 1 and 2). Interaction data was generated by Capture HiC.

E: Experimental strategy of RUNX1-ETO knockdown in Kasumi-1 cells. Transfection with either control siRNA or RUNX1-ETO siRNA on days 0, 2, 4, 6 or 8 in Kasumi-1 cells. Cells were collected for RNA, chromatin, DNase I or flow cytometry on designated days.

F: Left panel: Time course of DHS development after 2, 4 and 10 days of RUNX1-ETO depletion (see scheme in Figure S1E). Normalised tag counts (high: light blue, low, dark blue) are ranked alongside those of day 10 knock-down (KD) and control-specific (bottom), common and knock-down specific DHS are indicated on the left. Plotted alongside the same genomic coordinates are the distributions of AP1, C/EBP, ETS, RUNX1, E-box and CTCF motifs enriched in Kasumi-1 cells aligned to the summit of the day 10 DHSs (middle panel). The outermost right panels show ATAC-seq reads from normal hematopietic precursor cells (HSC, MPP, LMPP, CMP, GMP o or monocytes (Mono) from the corresponding interacting region plotted along-side.

G: Heatmap depicting genes differentially expressed during a time-course of 2, 4 and 10 days on RUNX1-ETO depletion ranked alongside up-and down regulated genes at day 10 of depletion.

H: RUNX1-ETO siRNA treatment in Kasumi-1 cells results in reduction in CD34 surface expression. Kasumi-1 cells after 2, 4 and 10 days of either RUNX1-ETO or control siRNA transfection were stained with CD34-PE and CD117 FITC. Representative flow cytometry plot of 3 independent experiments

I: Percentage of CD34+CD117+ cells. Mean of 3 independent experiments and error bars represent S.E.M. * denotes $p<0.05$ by paired t-test.

J: Principal component analysis with union of DHSs showing that the depletion of RUNX1-ETO leads to changes in the DHS profile of Kasumi-1 cells during 2, 4 and 10 days of RUNX1-ETO depletion.

K: Hierarchical clustering of Jaccard index/coefficient showing the similarity / differences in the footprint patterns.

L: Western blot analyses of protein extracts from: t(8;21) Kasumi-1 cells (transfected with control siRNA or RUNX1-ETO siRNA). Western blots were probed with either an anti-ETO, an anti-RUNX1 (N-terminal epitope antibody) and anti-C/EBPα, anti-LDB1, anti-LMO2, anti CTCF and anti-JUND antibodies. Anti-GAPDH antibody was used as a loading control. Representative western blot of 3 independent experiments

M: Fold-change of gene expression during the time course of RUNX1-ETO knock-down for selected genes

Figure S2

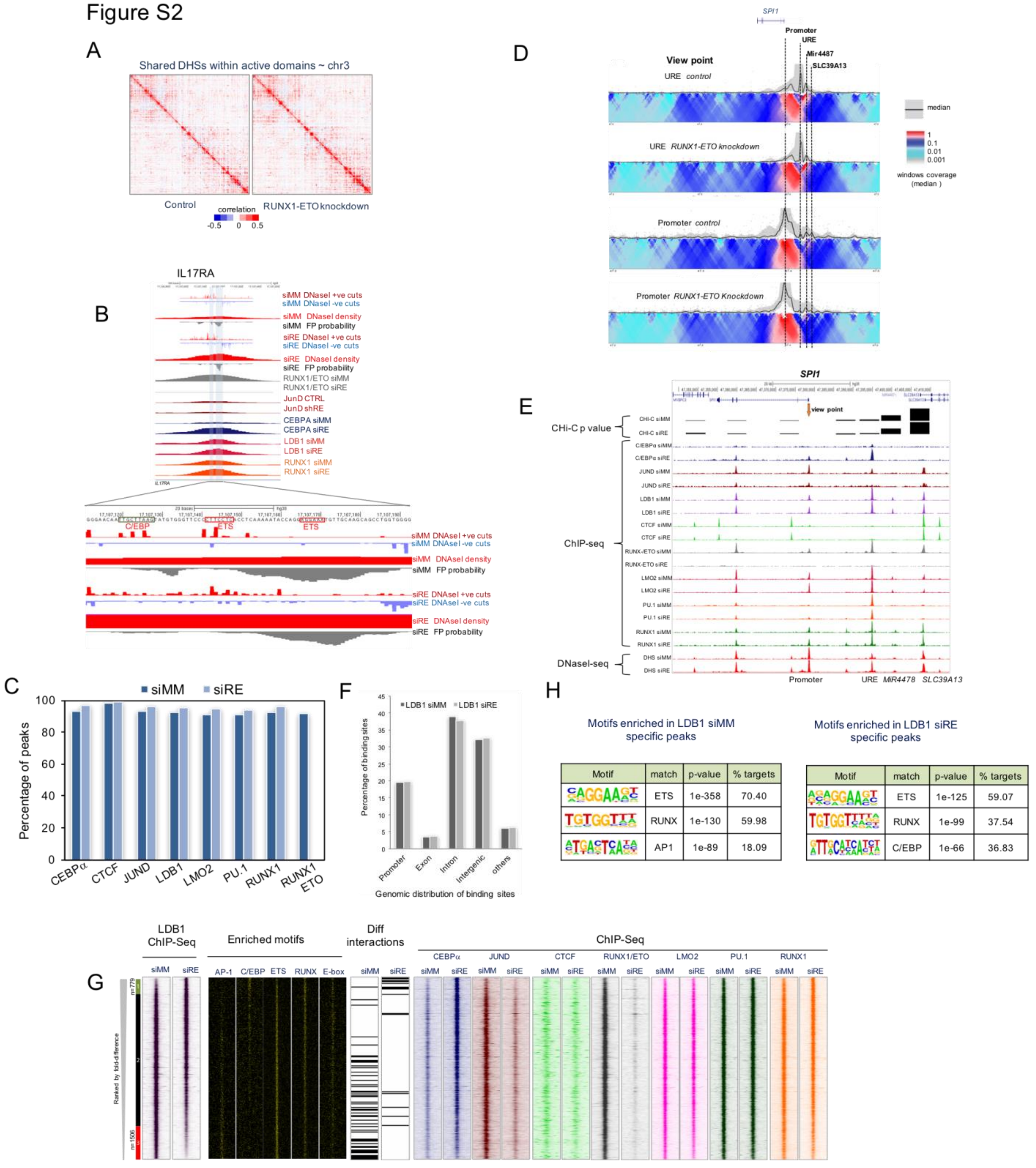

Supplemental Figure 2 (refers to Figure 2)

LDB1 participates in differential promoter-enhancer interactions after RUNX1-ETO depletion

A: DHS shared between siMM and siRE cells do not show changes in interactions. The heatmap represents the correlation of normalized interaction ratios across chr3 at 5 kb

resolution in DHS peaks shared between control and RUNX1-ETO depleted cells, the left panel shows the interaction heatmap for control and the right panel for knock-down cells. Each pixel represents a 5 kb section of the genome. Positive correlation is shown as red, negative correlation as blue.

B: UCSC genome browser screenshot of ChIP-seq and DHS data aligned with digital footprints at the IL17RA locus within a DHS shared between siMM and siRE samples. It also shows the binding pattern of RUNX1-ETO, JUND, C/EBPα, LDB1 and RUNX1 in Kasumi-1 cells as determined by ChIP. Footprint probabilities as calculated by Wellington (Piper et al., 2013) are indicated as gray columns below the lines. The bottom indicates the location of occupied ETS, and C/EBP motifs

C: Percentage of ChIP-seq high confidence peaks (Ptasinska et al., 2014) which show factor occupancy in digital footprint assays.

D: Validation of Chi-C data. The SPI1 locus does not show any differences in interactions before and after RUNX1-ETO depletion. Validation of Chi-C data and analysis of reproducibility. Local 4C contact profile with a viewpoint from the *SPI1* (PU.1) upstream regulatory element (URE) (upper two panels) and its promoter (lower two panels), using chromatin from Kasumi-1 cells transfected with control siRNA (siMM) RUNX1/ETO specific siRNA (siRE). In the top panel (main trend), the contact intensity (black line) is calculated using a running median analysis of normalized read counts with a 3 kb sliding window. The 20th and 80th percentile are visualized as a grey trend graph. In the bottom panel, contact intensities are computed using linearly increasing sliding windows (scaled 2–50 kb) and displayed as a color-coded heat map of positive 4C signal (maximum interaction set to 1). Local colour changes are log-scaled to indicate changes of statistical enrichment of captured sequences, corresponding to the enhancer-promoter interaction. The results presented here are an average of two biological replicates.

E: The SPI1 locus does not show any differences in interactions before and after RUNX1-ETO depletion. UCSC browser screenshot depicting interactions between the *SPI1* promoter and surrounding DHS. Interactions are depicted as bars abve the distance-dependent threshold level of statistical significance (CHi-C p-Value) together with the indicated ChIP and DHS data before and after RUNX1-ETO knock-down.

F: Graph showing the genomic distribution of LDB1 binding sites before and after RUNX1-ETO knock-down.

G: LDB1 ChIP-seq profiles ranked from top to bottom in order of decreasing relative DNA sequence tag count. Aligned to these coordinates are indicated enriched motifs and ChIP-

seq data. Column 1-2 represent the LDB-1 peaks from siMM or cells depleted of RUNX1-ETO for 2 days. Alongside, columns show enriched motifs for the TFs AP-1, C/EBP, ETS, RUNX1 and E-box in Kasumi-1 cells aligned to the summit of the LDB1 peaks. Then next columns show a binary representation of Capture HiC differential interactions aligned to the union of LDB1 peaks. Alongside, C/EBP, JUND, CTCF, RUNX1-ETO, LMO2, PU.1 and RUNX1 ChIP reads from Kasumi-1 cells with or without RUNX1-ETO depletion are plotted as indicated.

H: Results of de novo motif search of LDB1 specific peaks before and after RUNX1-ETO knock-down

Figure S3

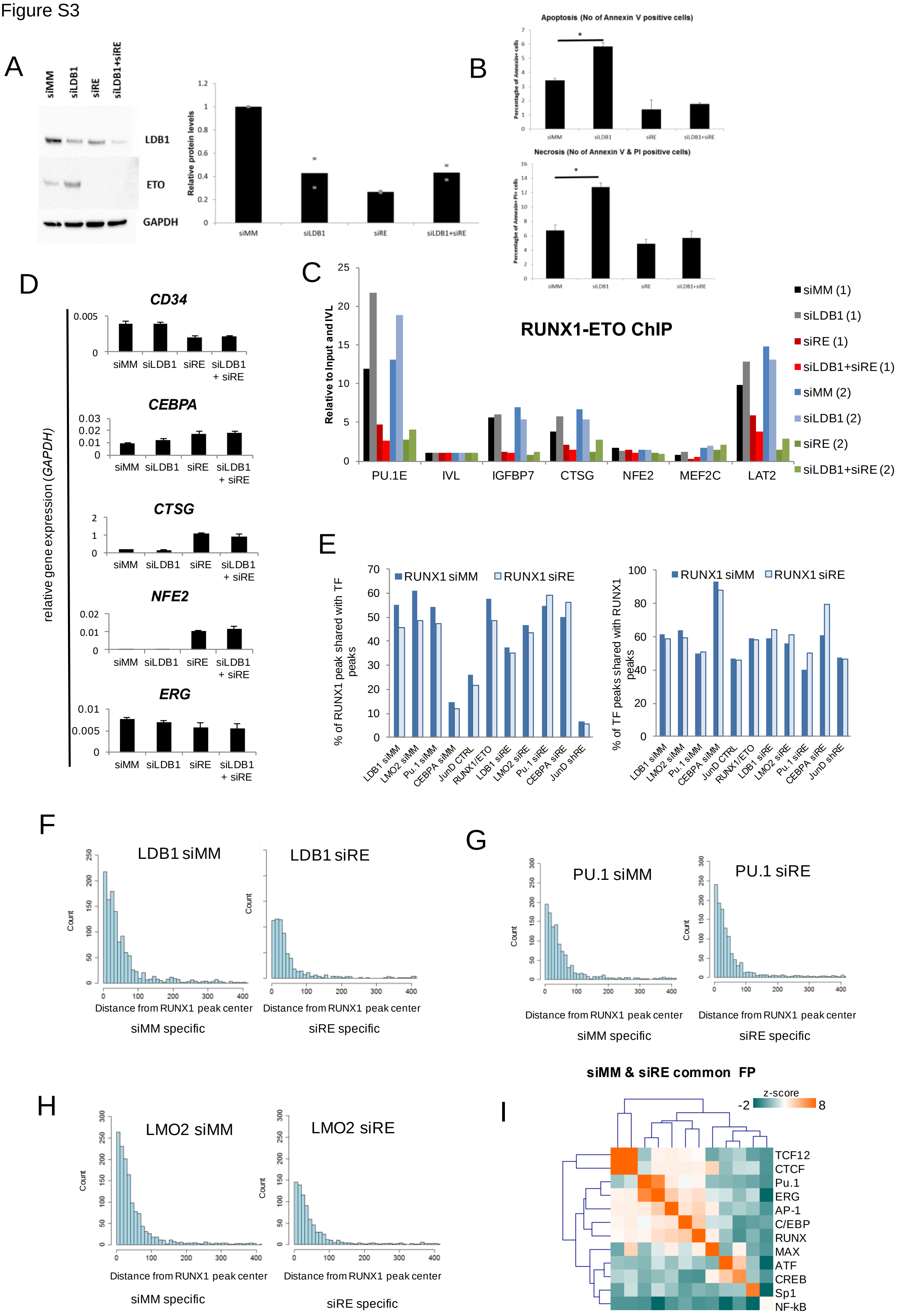
A
siMM
siLDB1
siRE
siLDB1+siRE
LDB1
ETO
GAPDH
Relative protein levels
B
Apoptosis (No of Annexin V positive cells)
Necrosis (No of Annexin V & PI positive cells)
C
RUNX1-ETO ChIP
Relative to Input and IVL
PU.1E
IVL
IGFBP7
CTSG
NFE2
MEF2C
LAT2
siMM (1)
siLDB1 (1)
siRE (1)
siLDB1+siRE (1)
siMM (2)
siLDB1 (2)
siRE (2)
siLDB1+siRE (2)
D
relative gene expression (GAPDH)
CD34
CEBPA
CTSG
NFE2
ERG
E
RUNX1 siMM
RUNX1 siRE
% of RUNX1 peak shared with TF peaks
% of TF peaks shared with RUNX1 peaks
LDB1 siMM
LMO2 siMM
Pu.1 siMM
CEBPA siMM
JunD CTRL
RUNX1/ETO
LDB1 siRE
LMO2 siRE
Pu.1 siRE
CEBPA siRE
JunD shRE
F
LDB1 siMM
LDB1 siRE
Count
Distance from RUNX1 peak center
siMM specific
siRE specific
G
PU.1 siMM
PU.1 siRE
H
LMO2 siMM
LMO2 siRE
I
siMM & siRE common FP
z-score
TCF12
CTCF
Pu.1
ERG
AP-1
C/EBP
RUNX
MAX
ATF
CREB
Sp1
NF-kB

Supplemental Figure 3 (refers to Figure 3)

Colocalization analyses highlight different combinatorial TF binding patterns in control and RUNX1-ETO depleted cells

A: Western blot indicating RUNX1-ETO and LDB1 protein expression levels in single- and double knock-down cells as indicated. An antibody against GAPDH was used as control. The bar plot showing quantification of relative LDB1 protein levels in single- and double knock-down as indicated. The graph on the right shows mean of 2 independent experiment, n=2

B: Knock-down of LDB1 leads to cell death**.** Annexin V and propidium iodide (PI) staining followed by flow cytometry analysis was used to measure cell viability at 10 days after treatment with siRNA. The percentage represents the sum of the proportion of cells positive for Annexin V (top panel) or Annexin V and PI (bottom panel). The graph shows the mean of 3 independent experiment with SD. * denotes $p<0.05$ by paired t-test

C: ChiP experiment showing that LDB1 depletion does not prevent RUNX1-ETO binding to distinct classes of RUNX1-ETO binding sites as indicated (n=2, with 2 independent experiments plotted individually)

D: LDB1 depletion does not alter the response to RUNX1-ETO knock-down. mRNA levels of the indicated genes 10 days after electroporation with the indicated siRNAs. The graph shows the mean of 3 independent experiment with SD

E: Bar plots show the percentage of Chip-peak overlap of other TFs with with RUNX1 peaks. The left plot shows the percentage of RUNX1 peaks that are shared with ChIP-seq peaks for other factors. The right plot shows percentage of ChIP-seq peaks that shared with RUNX1 peaks.

F, G: Bar plots illustrated the distribution of distances between RUNX1 ChIP-Seq peaks (before (left) and after (right) R/E KD) and LDB1 ChIP-Seq peaks peaks (E), PU.1 ChIP-Seq peaks (F) and LMO2 ChIP-Seq peaks (G).

I: C/EBP and AP-1 motifs do not co-localize in common DHS sites. Bootstrapping analysis of footprinted motifs for DNAseI common DHSs in Kasumi-1 cells. The heatmap shows the significance of co-localizing footprinted motifs clustering together as compared to sampling by chance.

Figure S4

A

Non-TF genes up-regulated by R/E depletion → Link to TF families via footprints → Gene regulatory network

B

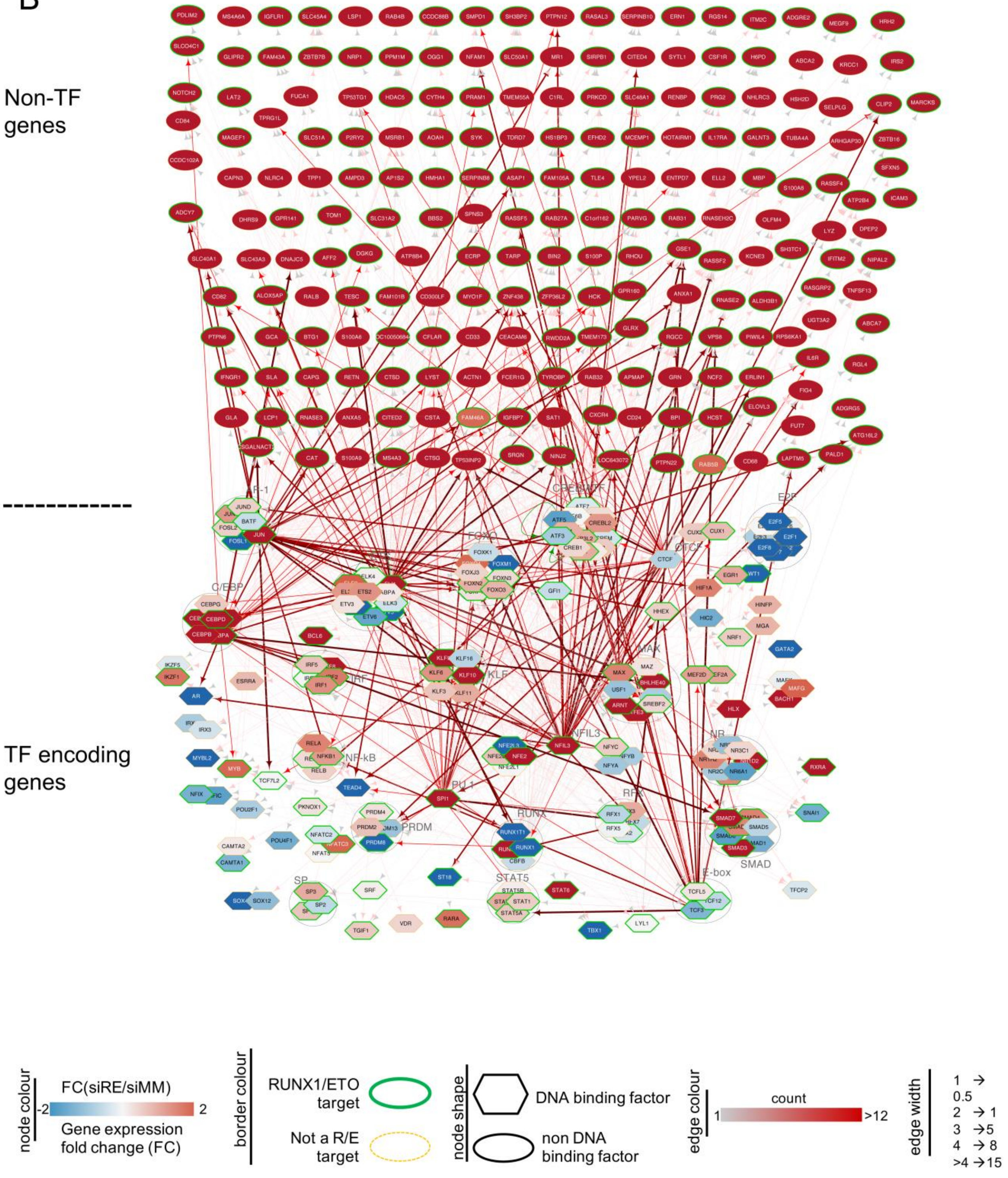

Node and edge attributes

Supplemental Figure 4 (refers to Figure 4)

Connections between factors and genes for genes at least two-fold up-regulated by RUNX1-ETO depletion

(A) Data analysis strategy. (B) Gene rgulatory network of all up-regulated (red) non-transcription factor (effector) genes after 10 days of RUNX1-ETO knock-down (upper rows) connected to genes encoding transcription factor families (lower rows) as determined by digital footprinting and CHi-C. Arrows going outwards can come from any TF family within a group, incoming arrows are specific for each gene. Bottom panel: node and edge attributes.

Suplemental Tables

motif	logo	motif	logo	motif	logo
AHR	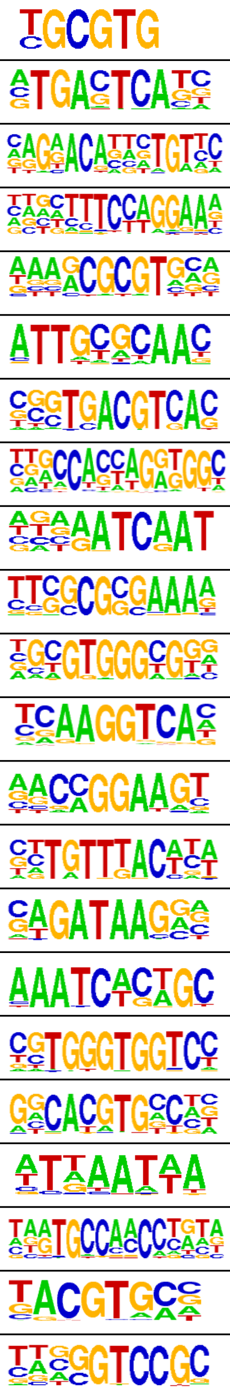	HSF1	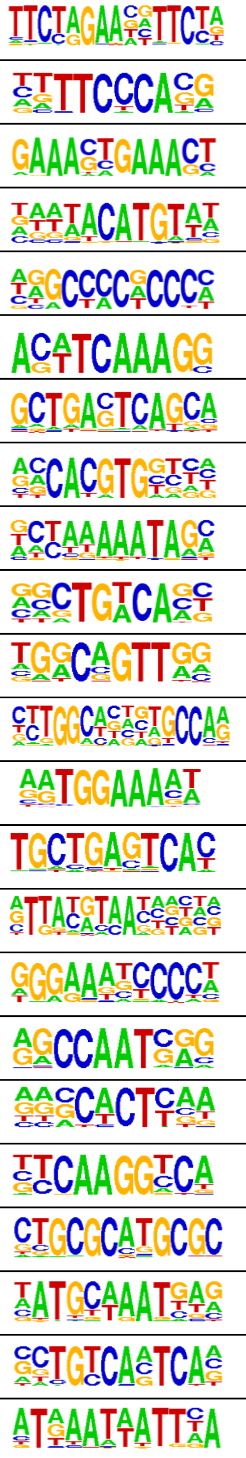	PRDM1	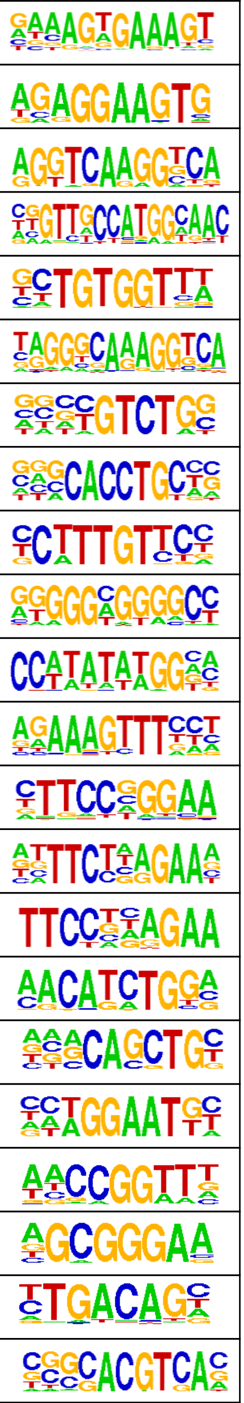
AP-1		IKZF		PU.1	
AR		IRF		RAR	
BCL6		IRX		RFX	
CAMTA		KLF		RUNX	
C/EBP		LEF1		RXR	
CREB/ATF		MAF		SMAD	
CTCF		MYC/MAX		SNAI	
CUT		MEF2		SOX	
E2F		MEIS		SP1	
EGR		MYB		SRF	
ESRRA		NF1		ST18	
ETS		NFAT		STAT3	
FOX		NFE2		STAT5	
GATA		NFIL3		STAT6	
GFI1		NF-kB		TAL1	
GLI		NFY		TCF3	
HES		NKX		TEAD	
HHEX		NR		TFCP2	
HIC1		NRF1		TFDP1	
HIF1A		OCT		TGIF	
HINFP		PKNOX1		XBP1	
		POU4F1			

Table S1. Curated position weight matrices used for motifs shared between transcription factor families as described in (Assi et al., 2018). Related to Figure 2, 3 and 4.

**Oligonucleotides**	**Forward**	**Reverse**
RUNX1-ETO siRNA	CCUCGAAAUCGUACUGAG AAG	UCUCAGUACGAUUUCGAG GUU
PU.1 14 3H (CHIP)	AACAGGAAGCGCCCAGTC A	TGTGCGGTGCCTGTGGTA AT
LAT2(CHIP)	*AAACCCAGAACAACCCAG GC*	ATGAGGAAGGATGTGTGT GCGG
CTSG(CHIP)	*TCAGTTGCTGCTGTGCTTC*	*TTCTCAATCCCCTGTCCCC AC*
NFE2(CHIP)	*AATAGCGAGGCCCCTCTT AG*	*ACCCAAACTGGAACACAA GG*
TBP (CHIP)	*CTGGCGGAAGTGACATTA TCAA*	*GCCAGCGGAAGCGAAGTT A*
CH18(CHIP)	*ACTCCCCTTTCATGCTTCT G*	*AGGTCCCAGGACATATCC ATT*
MEF2(CHIP)	*TTATGAGCTGAGCGTGCT GT*	*ATCTCACAGGTGCTGGCA TA*
IVL(CHIP)	*GCCGTGCTTTGGAGTTCTT A*	*CCTCTGCTGCTGCCACTT*
IGFBP7(CHIP)	*GTCAAGCACTAAAAGGAC AAACCG*	*TGAATGCCACTGGGAG*
GAPDH(cDNA)	*CCTGGCCAAGGTCATCCA T*	*AGGGGCCATCCACAGTCT T*
RUNX1/ETO(cDNA)	*TCAAAATCACAGTGGATGG GC*	*CAGCCTAGATTGCGTCTTC ACA*
CTSG(cDNA)	*TCCTGGTGCGAGAAGACT TTG*	*GGTGTTTTCCCGTCTCTGG A*
C/EBPα(cDNA)	*GAGGGACCGGAGTTATGA CA*	*AGACGCGCACATTCACATT*
CD34(cDNA)	*CACTGGCTATTTCCTGATG AAT*	*CCACCGTTTTCCGTGTAAT*
NFE2(cDNA)	*CCAAGGTGTGTTCAAAGA GGC*	*GGAGCCGAGTCAGGGAAG AC*
ERG(cDNA)	*ATGGAGGAGAAGCACATG CC*	*ATAGCGTAGGATCTGCTG GC*
Actin (short) (DNaseI)	GCAATGATCTGAGGAGGG AAGGG	AGCTGTCACATCCAGGGT CCTCA N/A
TBP promoter (short) (DNaseI)	CTGGCGGAAGTGACATTA TCAA	CCCGACCTCACTGAACCC
CH18 (short) (DNaseI)	AGGTCCCAGGACATATCC ATT	GTTCAAATTGTGTTTTGTG GTTA
Actin (long) (DNaseI)	GCAATGATCTGAGGAGGG AAGGG	GTGTCTTTCCTGCCTGAGC TGAC
TBP promoter (long) (DNaseI)	CTGGCGGAAGTGACATTA TCAA	GCCAGCGGAAGCGAAGTT A

CH18 (long) (DNaseI)	ACTCCCCTTTCATGCTTCTG	AGGTCCCAGGACATATCCATT
SPI1 promoter forward (4C-seq Primers)	AATGATACGGCGACCACCGAACACTCTTTCCCTACACGACGCTCTTCCGATCTAGTGAGGAGCAGTGGCGATC	CAAGCAGAAGACGGCATACGAACTATGCCCTGGCTCAGA
SPI1 URE forward (4C-seq Primers)	AATGATACGGCGACCACCGAACACTCTTTCCCTACACGACGCTCTTCCGATCTTGTCCAGGGAAGCCCAGATC	CAAGCAGAAGACGGCATACGATTCACCAGGCACAGACTT

Table S2. The list of PCR primers used for ChIP, gene expression, validating DNaseI digestion before DNaseI –Seq, viewpoint specific 4C-seq PCR primers. Related to STAR Methods.
